# Comparative transcriptome analysis between inbred and hybrids reveals molecular insights into yield heterosis of upland cotton

**DOI:** 10.1186/s12870-020-02442-z

**Published:** 2020-05-27

**Authors:** Kashif Shahzad, Xuexian Zhang, Liping Guo, Tingxiang Qi, Lisheng Bao, Meng Zhang, Bingbing Zhang, Hailin Wang, Huini Tang, Xiuqin Qiao, Juanjuan Feng, Jianyong Wu, Chaozhu Xing

**Affiliations:** 1 State Key Laboratory of Cotton Biology, Institute of Cotton Research of Chinese Academy of Agricultural Sciences, Key Laboratory for Cotton Genetic Improvement, Ministry of Agriculture, 38 Huanghe Dadao, Anyang, 455000 Henan China; 2 Jinhua Department of Economic Special Technology Promotion, Jinhua, 321017 Zhejiang China

**Keywords:** Cotton, Heterosis, RNA-Seq, Transcriptome, DEGs, Yield, Expression level dominance, QTLs, Co-expression

## Abstract

**Background:**

Utilization of heterosis has greatly improved the productivity of many crops worldwide. Understanding the potential molecular mechanism about how hybridization produces superior yield in upland cotton is critical for efficient breeding programs.

**Results:**

In this study, high, medium, and low hybrids varying in the level of yield heterosis were screened based on field experimentation of different years and locations. Phenotypically, high hybrid produced a mean of 14% more seed cotton yield than its better parent. Whole-genome RNA sequencing of these hybrids and their four inbred parents was performed using different tissues of the squaring stage. Comparative transcriptomic differences in each hybrid parent triad revealed a higher percentage of differentially expressed genes (DEGs) in each tissue. Expression level dominance analysis identified majority of hybrids DEGs were biased towards parent like expressions. An array of DEGs involved in ATP and protein binding, membrane, cell wall, mitochondrion, and protein phosphorylation had more functional annotations in hybrids. Sugar metabolic and plant hormone signal transduction pathways were most enriched in each hybrid. Further, these two pathways had most mapped DEGs on known seed cotton yield QTLs. Integration of transcriptome, QTLs, and gene co-expression network analysis discovered genes *Gh_A03G1024*, *Gh_D08G1440, Gh_A08G2210, Gh_A12G2183, Gh_D07G1312, Gh_D08G1467, Gh_A03G0889, Gh_A08G2199,* and *Gh_D05G0202* displayed a complex regulatory network of many interconnected genes. qRT-PCR of these DEGs was performed to ensure the accuracy of RNA-Seq data.

**Conclusions:**

Through genome-wide comparative transcriptome analysis, the current study identified nine key genes and pathways associated with biological process of yield heterosis in upland cotton. Our results and data resources provide novel insights and will be useful for dissecting the molecular mechanism of yield heterosis in cotton.

## Background

Cotton is derived from the Arabic word ‘quotn’ [1], belongs to the genus *Gossypium*, and has almost 50 species with diploid (2n = 26) and tetraploid (2n = 52) levels [2]. Among them, upland cotton (*G. hirsutum)* is allotetraploid, referred as new world cotton, and accounts for more than 90% of the world cotton production [3]. It has an exceptional yield, early maturity, and moderately good fiber qualities. Cotton is planted commercially for agricultural and industrial drives in the tropical and temperate region of the world on an area of 32–34 million hectares with total annual production of 25.65 million metric tons [4]. Based on the average of the last 3 years, cotton is one of the few major commodities whose global production more or less matches the world mill consumption. Staggered yield potential of recent genotypes and climate change are major constraints. The breeders should mitigate these problems by developing varieties or hybrids not only with superior yield and fiber quality but also with resistance against major pests, diseases and abiotic stresses. The commercialization of hybrid cotton in China started around 1980 and the planting area increased in subsequent years with the development of hybrid *Bt* cotton [5, 6].

Utilization of heterosis proficiently increased the quantity and quality of crops. Heterosis is a phenomenon in which offspring produce more superior characters than their parents [7, 8]. In the last century, EM East [9] distinguished that crosses between different allotetraploids in the same genus of *Nicotiana tabacum* formed exceptional heterosis. It was also experimentally observed by C Darwin [10] but Gorge. H Shull first time used the term heterosis in plant breeding [11]. After the introduction of hybrids, yield was raised effectively in many crops. Although cotton is an allopolyploid, it has more than two sets of basic chromosomes. Still, meaningful heterosis for different traits has been perceived in many filed experiments [12–15]. Cotton hybrid’s main impacts include self-sufficiency, stability in production, improved fiber quality, generation of employment, foreign exchange earnings, and development of seed industry [5]. Hybrids in cotton are developed through utilization of heterosis in two ways: First is conventional method that consists of emasculation and pollination with hands. Second is male-sterile system, which is an efficient method to reduce the cost of hand emasculation and ensure seed purity [16, 17].

The genetic basis of heterosis is perturbed and has been researched for almost a century using a variety of approaches, for instance, genetics [18, 19], molecular biology [20] omics [21], and physiological biochemistry [22]. Many researchers tried to explain crop heterosis with the so-called gene action hypothesis of dominance [23–25], overdominance [9, 26, 27], and epistasis [28, 29]. However, genetic diversity between two parents and level of heterosis is not simple and straightforward in cotton [30]. QTL mapping is advanced in 1990, makes some opportunities to understand individual QTLs, and interaction between them for heterosis. Genetic basis of heterosis is complicated, and involved dynamic dominance effect, epistasis and QTL by environmental interactions [31]. With the advancement in molecular research, many researchers concluded dominance, overdominance, and epistasis are basically conceptual and do not clarify the molecular mechanism and principals of heterosis [19, 32]. Meanwhile, it should be kept in mind that genetic models are equally important as the phenomenon of heterosis is a nonlinear effect from multiple heterozygous gene combinations. Moreover, yield traits are quantitative and many genetic characteristics function together to produce heterotic output, therefore a single genetic mechanism cannot explain genetic bases of heterosis in plants [33].

Considering the importance of F_1_ heterosis in breeding, the genetic and molecular mechanism of heterosis has extensively been investigated in agronomic crops like rice and maize with model biotechnological tools. For instance, it was observed in maize that incomplete dominance of deleterious alleles caused phenotypic trait variation and heterosis [34]. Allelic specific expression or imbalance expression of two parental alleles in hybrids contributes to heterosis in rice [35]. However, advance research with high throughput sequencing has not performed yet in cotton. The one reason behind this is the late availability of the whole genome sequence of upland cotton to the researchers [36, 37]. Further, the allopolyploid cotton genome has a low level of molecular polymorphism as compared to other crops [38] and many genome duplications events occurred before and after polyploidization resulted in natural gene silencing, organ-specific, and homoeologous biased gene expression in cotton [39]. A better understanding of the genetic mechanism of yield heterosis could enhance efficiency of future cotton breeding programs. Keeping in view the importance of heterosis and to accomplish the gap interlinked with molecular research in upland cotton, we designed a comprehensive study consisted of field experiments and genome-wide transcriptome analysis between F_1_ hybrids and their respective inbred parents. The following are the main objectives of this research: Selection of hybrids with consistence performance in yield traits in diverse environments. Detection of DEGs, mode of gene expression, and biological pathways critical for yield heterosis. Identification of putative candidate genes and overview of their regulatory mechanism with co-expression network analysis.

## Results

### Identification of cotton hybrids exhibiting different level of heterosis

**Fig. 1:**
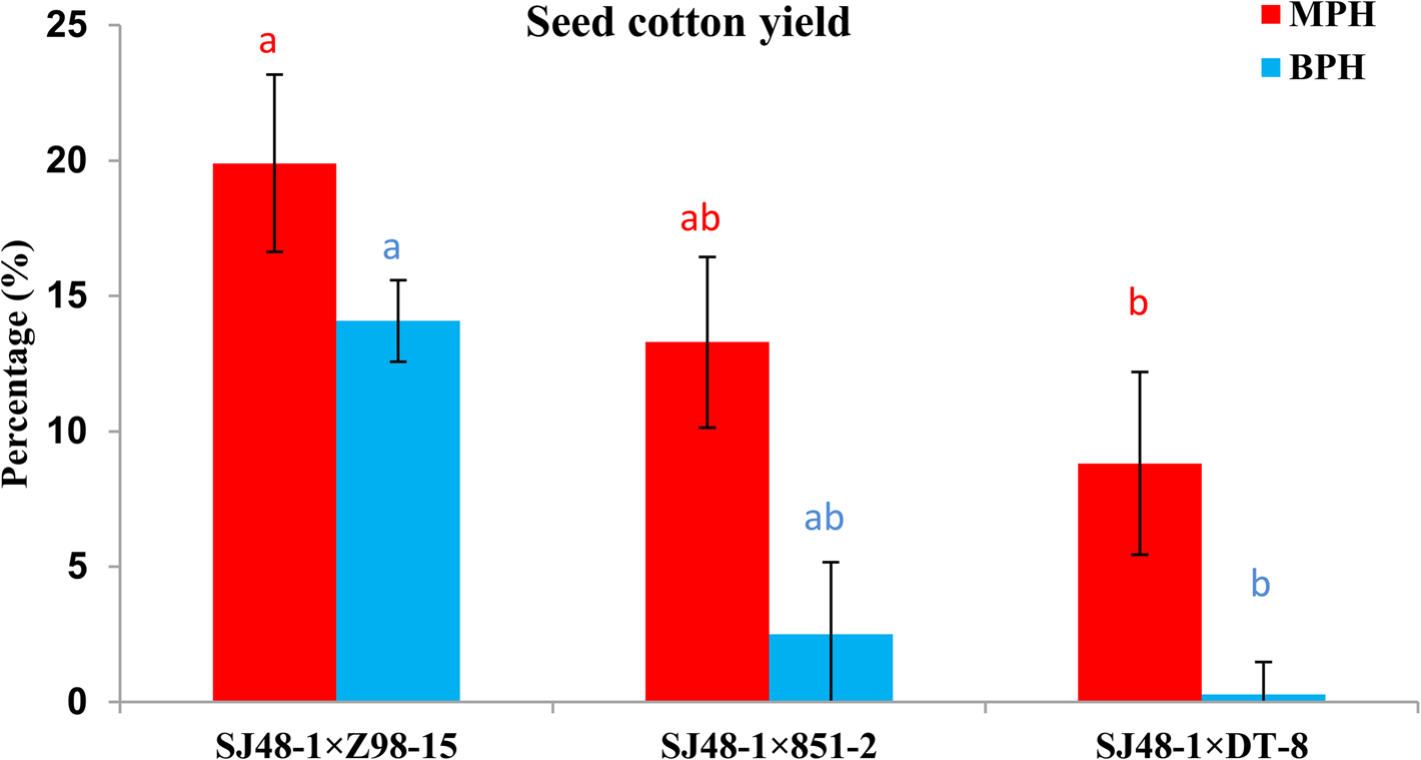
Percentage of mean seed cotton yield heterosis in high, medium, and low hybrids. SJ48–1 × Z98–15 SJ48–1 × 851–2 and SJ48–1 × DT-8 correspond to high, medium, and low hybrids, respectively. Different letter represents significant difference among mean within same group at *p < 0.05*

Our previous study of 11 inbred lines and 30 intraspecific hybrids in six environments found that cotton hybrids had better and stable performance compared to inbred lines in yield traits [40]. Here, analysis of heterosis in these hybrids showed majority of hybrids had positive mid parent heterosis (MPH) and better parent heterosis (BPH) for boll weight, seed cotton yield (SCY), lint yield and lint percentage (Additional file 14: Table S1). Later on, three hybrids with different level of yield heterosis were identified and defined as high (H), medium (M) and low (L). Notably, H-hybrid (SJ48 × Z98) produced 19.9% MPH and 14.1% BPH in SCY (Fig. 1). M-hybrid (SJ48 × 851) had MPH of 13.3% and BPH of 2% for SCY, whereas L-hybrid (SJ48 × DT) showed the only MPH of 8.8% for SCY. These three hybrids had common maternal inbred parent but paternal inbred parents all differed. H-hybrid is cross of maternal line SJ48 (A) with paternal parent Z98 (B) and M-hybrid with paternal parent 851 (C), whereas L-hybrid is cross of same maternal line with paternal parent DT (D). The differences in the level of yield heterosis suggest that these three hybrids together with their inbred parents are suitable for studying comparative transcriptome analysis and regulatory mechanism of yield heterosis in cotton.

### Transcriptome profiles of 63 RNA libraries of cotton hybrids and their inbred parents at squaring stage

To understand global transcriptome profiles of yield heterosis in cotton, three contrasting hybrids and their four inbred parents were used to perform RNA sequencing at the squaring stage. Leaf (Hereafter RL), flower buds (F), and 1 day post anthesis (1 DPA) ovule tissues with three biological replications were used for each genotype. In total, 63 libraries (7 × 3 × 3) were constructed for deep Illumina pair-end RNA sequencing. A total of ~ 43–50 million reads were generated per library (Additional file 15: Table S2). The average percentage of the valid read was ≥98.5%. The value of Q30% was above 95% in this sequencing. Approximately, 94.9% of clean reads were mapped to the reference *G. hirsutum* genome [36]. Almost 90.5% reads were mapped to exon region in each sample and genotype. Wherein, intron and intergenic region mapping were ≤ 5% (Additional file 1: Figure S1). The brief detail of total mapped, unique mapped, multi mapped, non-splice, and splice reads for each library can be seen in Additional file 15: Table S2. The principal component analysis confirmed variation among tissues and genotypes in this experiment (Additional file 2: Figure S2). Two biological replicates were concentrated in same cluster in biplot. Pearson correlation test for all replicate and genotypes exposed RL tissue had a strong correlation among each other (≥75) and weak with F and 1 DPA (≤ 50) (Additional file 3: Figure S3). Correlation between F and 1 DPA samples was relatively strong (~ 55–60).

### Global transcriptome changes for cotton hybrids and their inbred parents

**Fig. 2:**
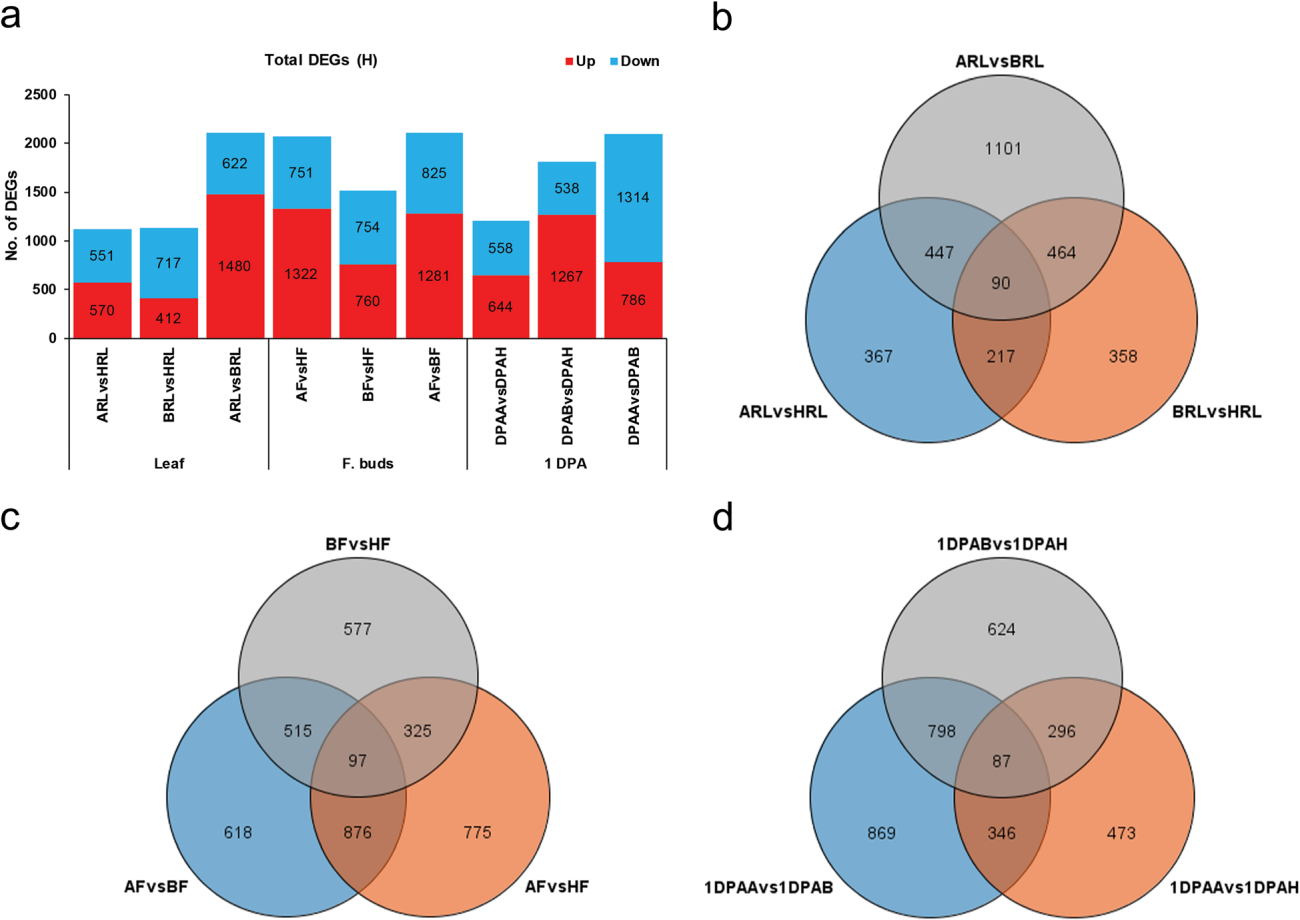
Total DEGs and their distribution in high-hybrid parent triad. **a** shows total number of DEGs in the hybrid parent triad. **b**, **c**, and **d** respectively represent distribution of unique and common DEGs in leaf (RL), flower buds (F) and 1 DPA ovule. Maternal parent is denoted with A, paternal parent with B, and high hybrid with H in each figure

The number of total expressed genes provides an overview of the transcriptomic landscape for all datasets. The gene was considered to be expressed, if a gene has expression higher than zero in all three biological replicates of a sample. A total of ~ 60,000 genes were expressed in each dataset (Additional file 4: Figure S4). Total number of expressed genes was much higher in flower buds compared to leaf and 1 DPA ovule. Different expression level for mRNA calculated as fragments per kilobase of exon model per million reads mapped (FPKM) was used to analyze the dynamic changes of transcriptomes between hybrids and their respective parents. Genes with expression levels higher than 0.5 FPKM in at least in one sample of each tissue were used for further analysis. The differentially expressed genes (DEGs) between samples were selected with log2 (fold change) > 1 or < − 1 and with statistical significance (*p-value < 0.05*). The comparison of transcriptomes was performed for each hybrid parent triad and tissue. Total number of DEGs (Up + down) and their distribution among H-hybrid parent triad is shown in Fig. 2. Comparative analysis among H and its maternal parents (A) showed highest number of total DEGs in flower buds (F), while compared with paternal parent (B), highest DEGs were respectively identified in 1 DPA (Fig. 2a). Combination of both parents A and B displayed higher number of DEGs (~ 2100 in all tissues) relative to H-hybrid. The results of DEGs distribution revealed that major portion of genes were common, whereas less were unique in each tissue (Fig. 2b, c, d). For instance, the combination of A with H had only 367 unique DEGs in RL.

**Fig. 3:**
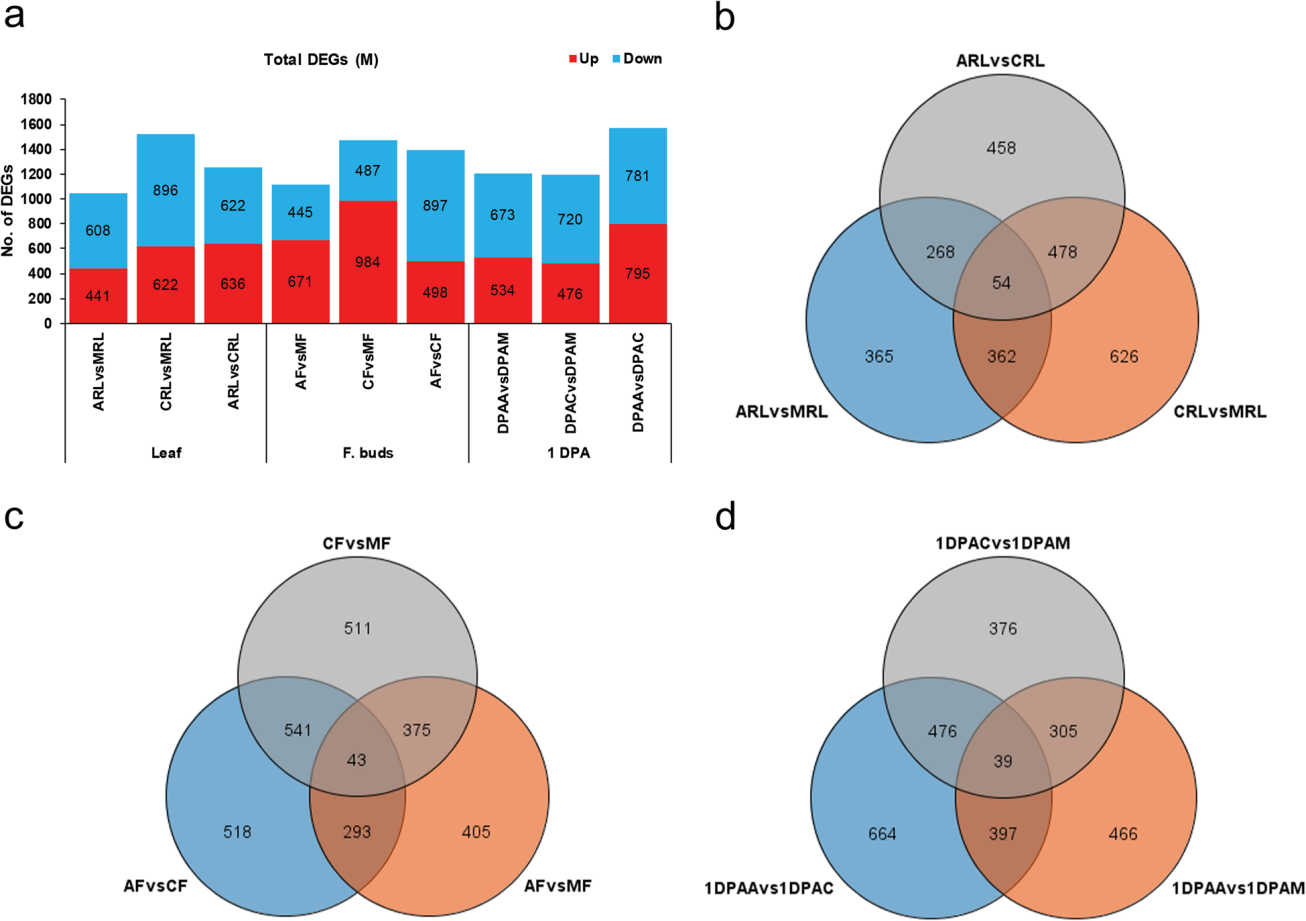
Total DEGs and their distribution in medium-hybrid parent triad. **a** shows total number of DEGs in hybrid parent triad. **b**, **c**, and **d** respectively represent distribution of unique and common DEGs in leaf (RL), flower buds (F) and 1 DPA ovule. Maternal parent is denoted with A, paternal parent with C, and medium hybrid with M in each figure

**Fig. 4:**
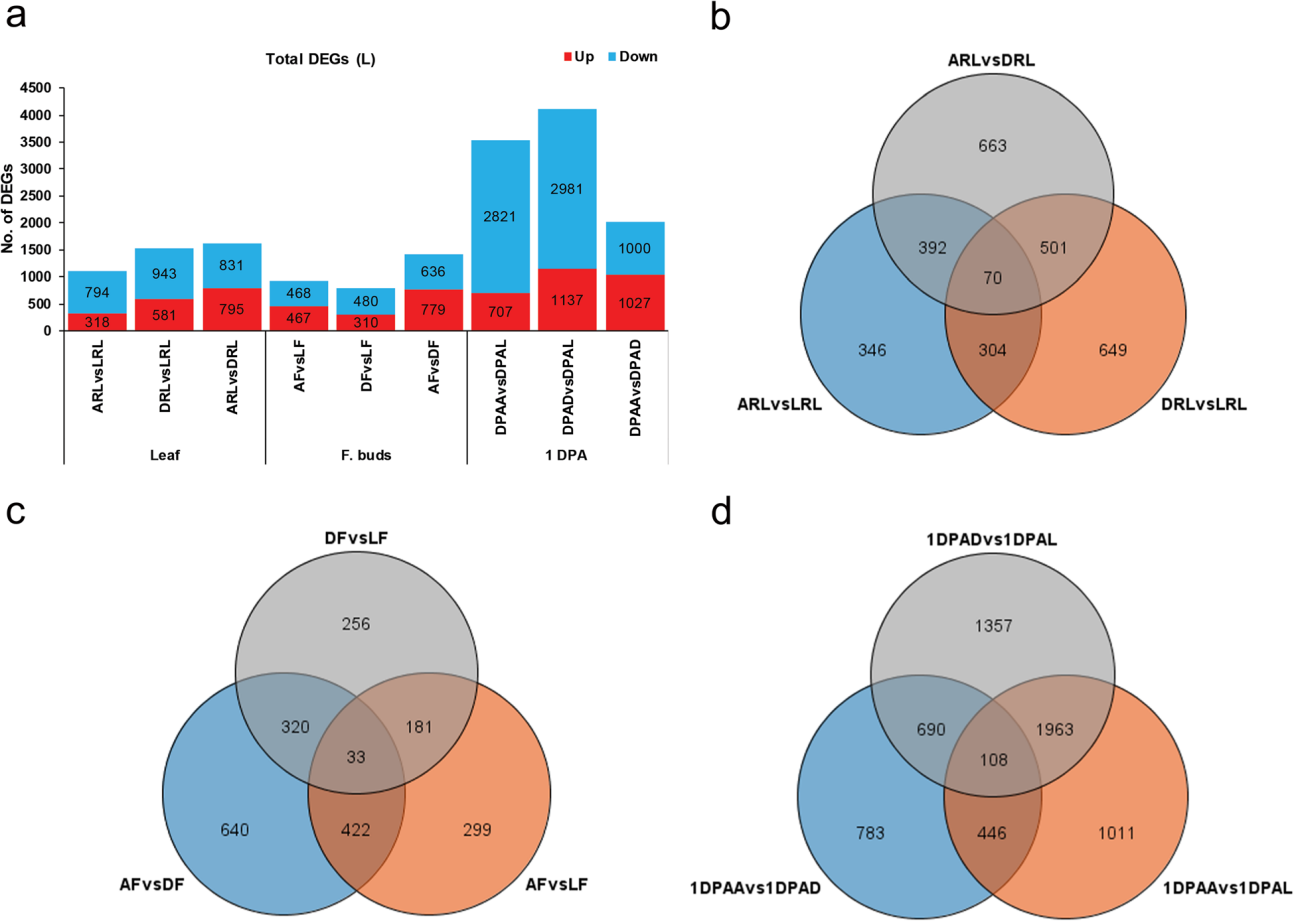
Total DEGs and their distribution in low-hybrid parent triad. **a** shows total number of DEGs in hybrid parent triad. **b**, **c**, and **d** respectively represent distribution of unique and common DEGs in leaf, flower buds and 1 DPA ovule. Maternal parent is denoted with A, paternal parent with D, and low hybrid with L in each figure

The results revealed that M with its maternal parent (A) had lower percentage of DEGs as compared with paternal parent (C) (Fig. 3a). Further, more common and less unique DEGs were identified in each tissue (Fig. 3b, c, d). Comparative analysis of DEGs for L-hybrid parent triad is summarized in Fig. 4. The result showed that L versus maternal parent (A) had 1112 total DEGs in RL, 935 in F, and 3528 in 1 DPA (Fig. 4a). The comparison of L and paternal parent (D) showed 1524, 790, and 4118 total number of DEGs in RL, F, 1 DPA, respectively. Comparison between parent A and D displayed higher number of DEGs in 1 DPA. Additionally, the results of distribution of DEGs in each tissue were similar to high and medium hybrids (Fig. 4b, c, d). Comparative transcriptome analysis in each hybrid parent triad at squaring stage determined that percentage of genetic differential expressions in hybrids was similar to those of between parents.

### Analysis of expression level dominance for hybrids

**Fig. 5:**
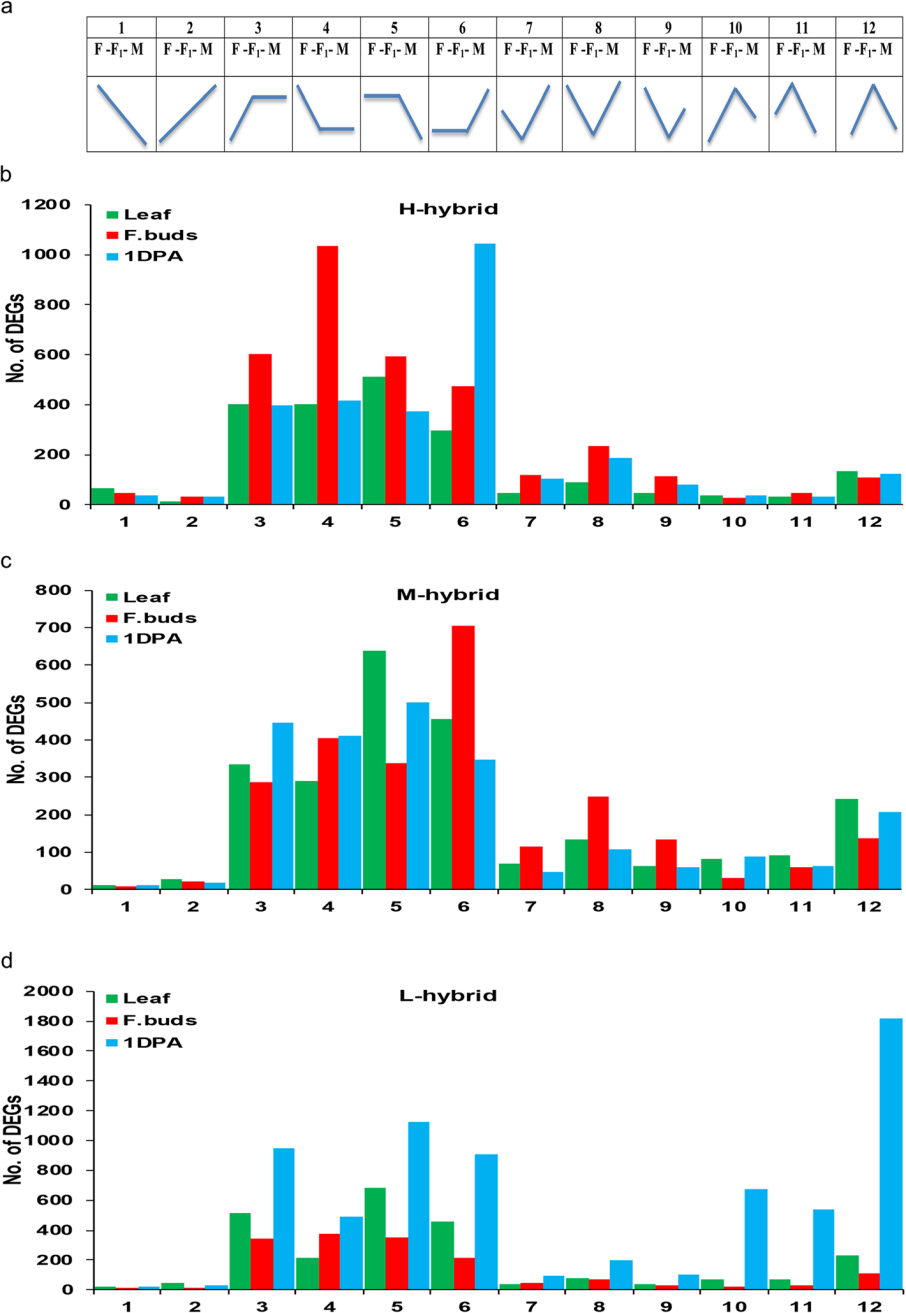
The gene expression groups in each F_1_ hybrids. **a** Expression patterns of 12 groups, M: paternal parent, F_1_: Hybrid, and F: maternal parent. **b**, **c**, and **d** indicate total number of genes in each group of high (H), medium (M), and low (L) hybrids, respectively

Expression level dominance is a phenomenon in which offspring follow the expression patterns one of two diploids parents. In order to identify the expression magnitude and directionality in interspecific F_1_ cotton hybrids, DEGs were divided into 12 possible groups as described in material and methods following the conventions of Yoo et al.*,* [41] (Fig. 5a). Results showed that the magnitude of expression of most genes in hybrids was similar to that of one parent (Fig. 5). For instance, most of the genes in leaf and 1 DPA ovule of H-hybrid displayed higher-maternal dominance expression and lower-maternal dominance expression respectively (Fig. 5b; Additional file 5: Figure S5a, c). Lower-paternal dominance expression had highest proportion in flower buds (Fig. 5b; Additional file 5: Figure S5b). Non-additive expressed genes in leaf and 1 DPA ovule of M-hybrid mostly displayed higher-maternal dominance expression (Fig. 5c; Additional file 6: Figure S6a, c). Wherein lower-maternal dominance expression had highest proportion in flower buds (Fig. 5c; Additional file 6: Figure S6b). In case of L-hybrid, most of leaf genes showed higher-maternal dominance expression (Fig. 5d; Additional file 7: Figure S7a), while lower-paternal dominance expression had highest portion in flower buds (Fig. 5d; Additional file 7: Figure S7b). Most of the genes in 1 DPA ovule displayed higher maternal dominance expression in this hybrid (Fig. 5d; Additional file 7: Figure S7c). In accordance with analysis of expression level dominance, most DEGs of hybrids displayed parent expression level dominance (P-ELD) at squaring stage in different tissues that probably play role for yield heterosis of cotton.

### Functional annotation and pathway enrichment analysis of DEGs with P-ELD

To identify the function of DEGs with P-ELD, a total of four gene sets (3–6 groups) were pooled to perform GO and KEGG enrichment analysis for every hybrid and tissue (Additional file 16: Tables S3 and Additional file 17: Tables S4). GO enrichment analysis for H-hybrid showed majority of genes in each tissue had functional annotation related to membrane, ATP, and protein binding (Additional file 8: Figure S8a). Genes associated with chloroplast, extracellular region, and ATP binding had highest portion in leaf of M-hybrid (Additional file 8: Figure S8b). In flower buds, most of the genes were linked with biological process, molecular function, and integral component of membrane. Most abundant functional gene groups in 1 DPA ovule were biological process, chloroplast, and mitochondrion (Additional file 8: Figure S8b). Most enriched GO terms in leaf of L-hybrid were ATP binding and serine/threonine kinase activity (Additional file 8: Figure S8c). Functional annotation related to biological process, integral component of membrane, and chloroplast were abundant in flower buds, whereas gene involved in plasma membrane, regulation of transcription, and mitochondrion had highest portion in 1 DPA ovule of L-hybrid (Additional file 8: Figure S8c).

**Fig. 6:**
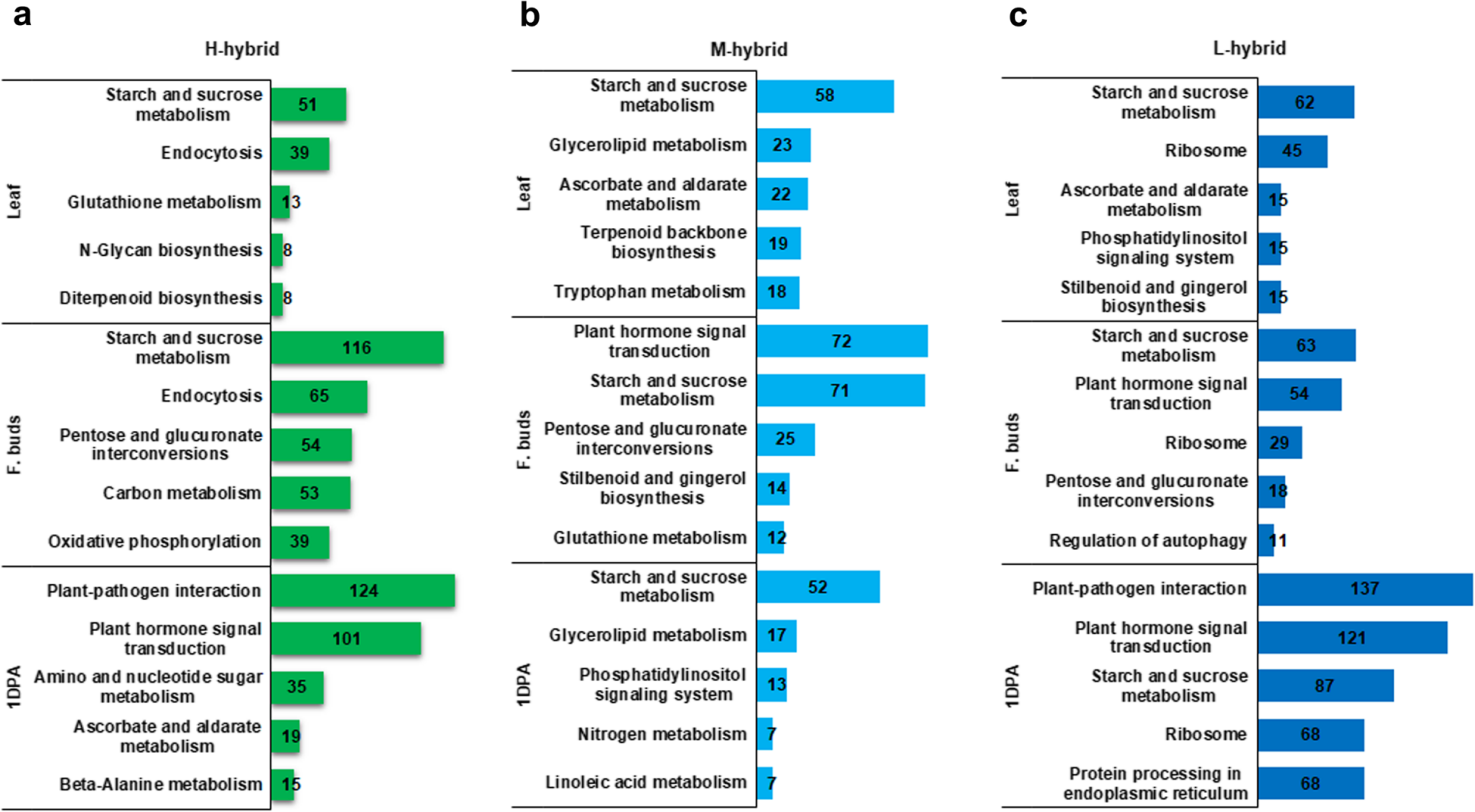
Most enriched pathways for DEGs with parents like expression in hybrids. **a, b,** and **c** represent pathway analysis in leaf, flower buds and 1 DPA ovule, respectively

KEGG enrichment analysis of P-ELD genes exposed that starch and sucrose metabolism, endocytosis, and tryptophan metabolism had more significance in leaf of H-hybrid (Fig. 6a). Majority of genes in flower buds were enriched in starch and sucrose metabolism, phagosome, and pentose and glucuronate interconversions. Whereas plant-pathogen interaction and plant hormone signal transduction were more representative pathways in 1 DPA ovule (Fig. 6a). Leaf of M-hybrid showed more enrichment in metabolic pathways e.g., starch and sucrose, ascorbate and aldarate, and tryptophan (Fig. 6b). In flower buds, starch and sucrose metabolism and plant hormone signal transduction had more gene enrichment (Fig. 6b). Starch and sucrose metabolism and phosphatidylinositol signaling system was enriched and significant in 1 DPA of this hybrid (Fig. 6b). Pathway categorization showed that L-hybrid had starch and sucrose metabolism and ribosome significance in leaf (Fig. 6c), whereas starch and sucrose metabolism, plant hormone signal transduction, and ribosome were more representative pathways in flower buds (Fig. 6c). In 1 DPA ovule, plant hormone signal transduction, plant-pathogen interaction, and starch and sucrose metabolism had more gene enrichment in this hybrid (Fig. 6c).

The common function of P-ELD genes in hybrids was integral component of membrane, biological process, mitochondrion, and ATP binding (Additional file 9: Figure S9a). Genes involved in common pathways such as sugar metabolism, plant hormone signal transduction, and wax biosynthesis had more significance in hybrids growth during reproductive stage (Additional file 9: Figure S9b). The nutrient assimilation and their distribution have key importance in plants during reproductive growth. Theoretically, the difference in genes expression involved in biological process of hormone and sugar metabolite may contribute to the yield heterosis of cotton.

### Mapping of key pathways DEGs on known seed cotton yield QTLs

**Fig. 7:**
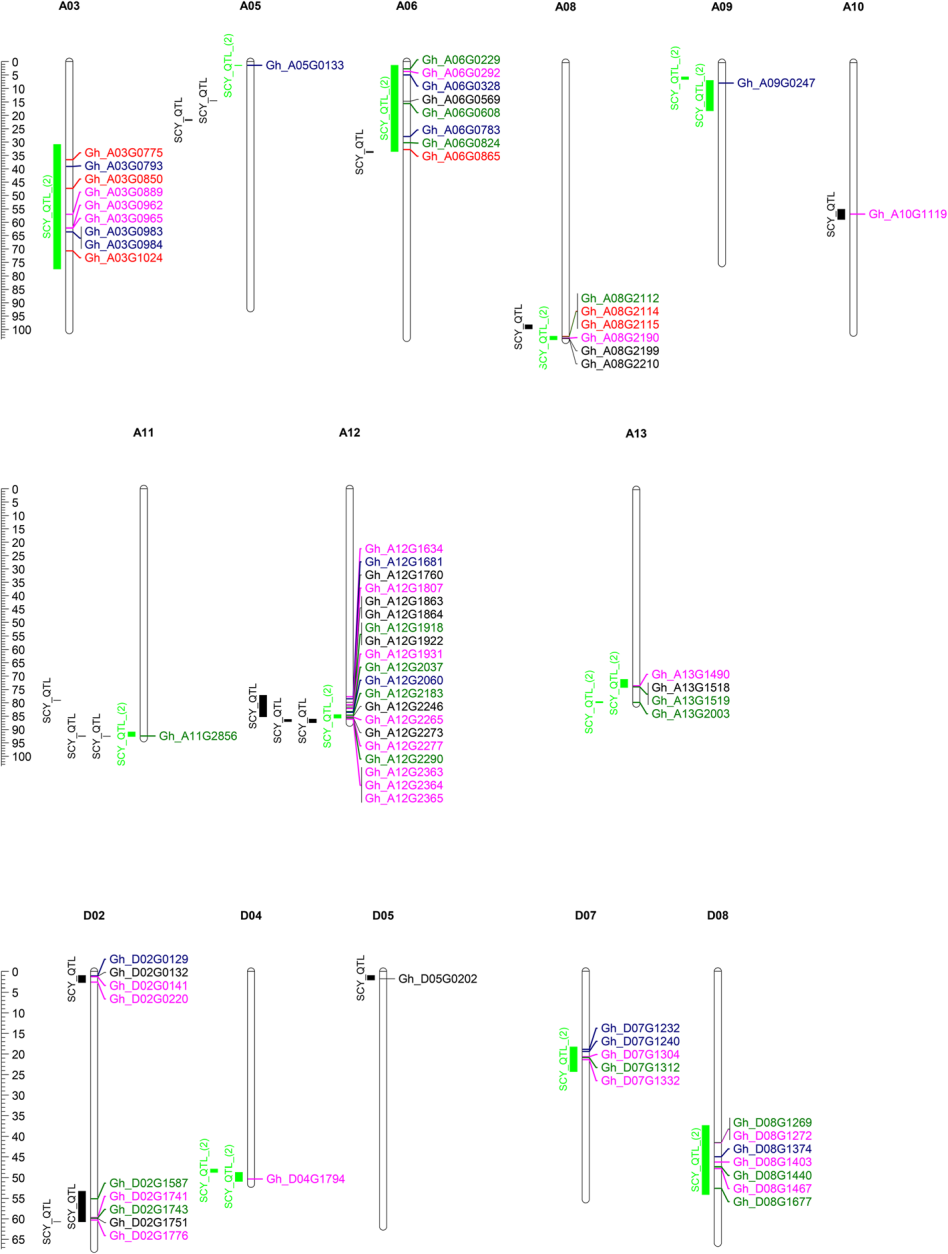
Distribution of hybrids DEGs with parents like expression on known seed cotton yield QTLs. Green, blue, and pink colors respectively indicate high, medium, and low hybrid-specific DEGs. Red and black colors show common DEGs among all and any of two hybrids, respectively. QTLs region reported more than once is in green color

QTL provide associations between genomics and phenomics and using QTLs can be an effective approach to understand the genetic complexities of yield heterosis [42, 43]. Here, we investigated the relationship between key pathway DEGs, seed cotton yield QTLs, and yield heterosis. Hybrids DEGs involved in key pathways were used to map on already known 57 seed cotton yield (SCY)_QTLs (Additional file 18: Table S5). Mapping results showed that 74 hybrid DEGs were mapped on 43 QTLs (Fig. 7). Interestingly, most of the genes were mapped on QTLs regions that were reported more than once (Fig. 7). Out of these DEGs, 6 genes had differential expression in all hybrids compared to their parents, whereas 13 were common between any of two hybrids. 13 DEGs were specific to medium hybrid. However, 17 and 25 DEGs were specific to high and low hybrid respectively (Additional file 10: Figure S10).

### Co-expression network analysis of high and low hybrids

Weighted gene co-expression network analysis (WGCNA) is an important tool for inferring the potential relationships between co-expressed genes, construction of co-expression network helps to understand the functional linkage between gene groups instead of individual genes, and provides novel insight into the system-level understanding of a biological process [44, 45]. Here, co-expression network analysis was performed in high and low hybrids by using DEGs (Pooled from leaf, flower buds, and 1 DPA) identified in our RNA-seq data analysis. Highly connected genes were clustered into distinct modules. Cluster dendrogram displayed that interconnectivity and size of each module was quite different in high and low hybrids (Additional file 11: Figure S11a, b). Interestingly, 8862 genes distributed in 21 modules with the size ranging from 34 to 1972 genes were identified in high hybrid, wherein 7863 genes scattered in 33 modules with the module size ranging from 42 to 2880 were determined in low hybrid (Additional file 19: Table S6). The correlation analysis between high and low hybrids modules was very strong (Additional file 11: Figure S11c). In high hybrid, average connectivity of genes in many modules was higher as compared to low hybrid (Additional file 11: Figure S11d). Although turquoise and blue module had the highest connected genes in both hybrids, but correlation coefficients among different genes were much higher in blue module.

### Genes regulatory co-expression network for seed cotton yield heterosis

**Fig. 8:**
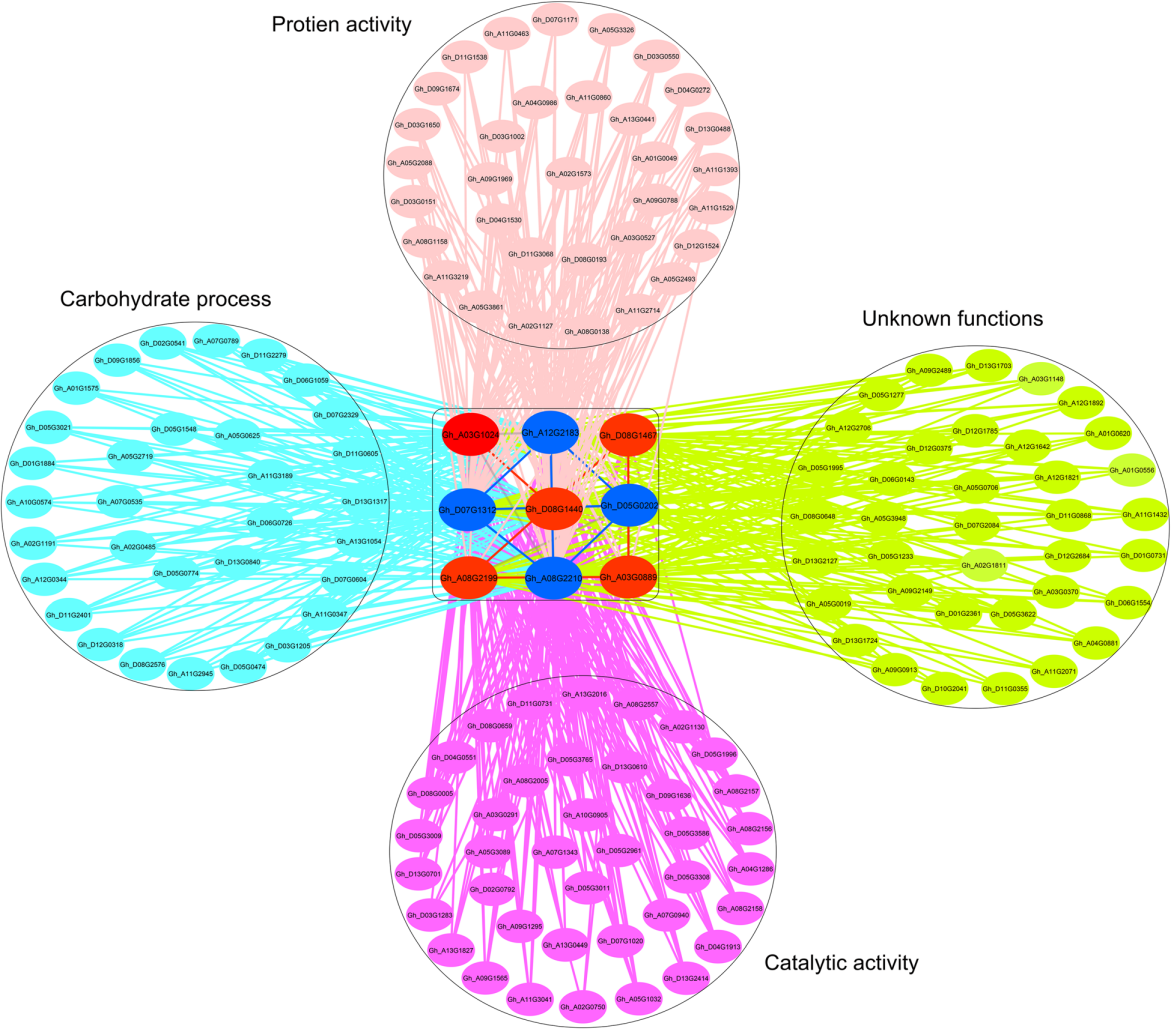
The co-expression network of known seed cotton yields QTLs mapped nine genes. Red and blue nodes respectively represent high and low hybrid-specific genes. Gene to gene correlation weight is above 0.40 in this network

To construct the significant regulatory network for yield heterosis, first, we selected blue gene module in both hybrids. Secondly, the DEGs mapped on SCY-QTLs together with their interaction genes having weight ≥ 0.40 were screened (Additional file 20: Table S7). Subsequently, our constructed gene co-expression network showed that nine SCY-QTLs genes had many interconnected genes involved in biological and molecular functions such as carbohydrate metabolic processes, catalytic activities, and protein binding or transportation (Fig. 8). We considered these nine genes as putative candidate genes for yield heterosis. These genes showed hybrid specific expression profile in RNA-seq analysis (Additional file 12: Figure S12). Out of nine genes, two genes *Gh_A03G1024* (*BZR1*: Brassinazole-resistant 1) and *Gh_D08G1440* (*ASK8*: Shaggy-related protein kinase theta) involved in plant hormone signal transduction pathway showed differential expressions in flower buds of high hybrid. Another three starch and sucrose metabolism pathway also had differential expressions in flower buds of high hybrid. These genes were *Gh_A08G2210* (Endoglucanase 16), *Gh_A12G2183* (*GBSS1*: Granule-bound starch synthase 1, chloroplastic/amyloplastic), and *Gh_D07G1312* (*APL2*: Glucose-1-phosphate adenylyltransferase large subunit 2, chloroplastic). The remaining four had specific differential expression in 1 DPA ovule of low hybrid. Three of them denoted as *Gh_D08G1467* (*MPK4*: Mitogen-activated protein kinase 4), *Gh_A03G0889* (*PHO1*: Phosphate transporter PHO1 homolog 3), and *Gh_A08G2199* (*JAZ10*: Jasmonate-zim-domain protein 10) were enriched in plant hormone signal pathway. The remaining one gene *Gh_D05G0202* (*CRR21*: Pentatricopeptide repeat-containing protein) belonged to starch and sucrose metabolism pathway. Collectively, gene co-expression network analysis in hybrids not only helped to screen key genes but also provided novel insights into the regulatory mechanism of yield heterosis of cotton.

### Quantitative real-time PCR (qRT-PCR)

We selected nine candidate DEGs to perform qRT- PCR analysis. These genes showed hybrid and tissue-specific expression in RNA-seq, mapped on SCY-QTLs, and had the highest interconnected genes in the co-expression network. Primer was designed in gene-specific way to ensure accuracy of expression (Additional file 21: Table S8). Analysis results showed *Gh_A03G1024*, and *Gh_D08G1440*, *Gh_A12G2183, Gh_A03G0889, Gh_D05G0202, Gh_A08G2199, Gh_D08G1467* and *Gh_D07G1312* displayed a significant change in each hybrid compared from their one parent or both parents (Additional file 13: Figure S13). However, gene *Gh_A08G2210* showed no statistical difference in hybrids. More interestingly, these genes apparently showed the expression similar to their maternal or paternal parent as determined in RNA-seq results.

## Discussion

### Usefulness of cotton hybridization and overview of transcriptome sequence

The utilization of heterosis has long been one of the main objectives of cotton breeders. Both intraspecific and interspecific cotton crosses exhibited meaningful heterosis for yield and yield-related traits [12, 15, 46–48]. However, hybridization in intraspecific crosses of upland cotton requires more efforts to obtain significant positive heterosis. Our field experimentation at six different environments revealed more than 70% of intraspecific hybrids of upland cotton had positive MPH for yield traits in each environment. The level of heterosis may be decreased in next generations. However, a previous research found both F_1_ and F_2_ crosses in cotton out-yielded their parent by 20–40% and 6–10%, respectively [49]. Other reported a MPH of 20% in F_1_ and 10% in F_2_ for lint yield [50]. Some researcher witnessed upland cultivars displayed higher MPH than modern cultivars in lint yield [51]. The level of heterosis in any desired trait depends on hybridized parents and breeding design. Selection of genetically superior and physiological effective parents already improved maize yield up to 15% in the USA [32] and 10–20% rice in the China [52].

To better understand the genetics behind yield heterosis, transcriptome sequencing at the start of the reproductive stage was performed using three contrasting yield hybrids with their four inbred parents. Comparative analysis of DEGs in all datasets identified total number of DEGs in hybrids was similar to those of between parents. Additionally, few unique genomic differences existed in hybrids compared to parents at the gene expression level. These results illustrate transcriptional reprogramming following hybridization even with few quantitative or qualitative differences in gene expression may lead to phenotypic variation in hybrids. Furthermore, some genes in hybrids may expressed differently and perform function better in some tissues or conditions, while others may be superior in other tissues or conditions. Accumulative effect of these few genes may derive heterosis in hybrids. Research in many agronomic crops has confirmed hybridization has a dramatic effect on genes expressions [53–56]. Previously, comparative analysis between hybrids and their parents reported only 0.8–2.3% DEGs in *B. napus* [56] and 10.6% in super hybrid rice LYP9 [57]. Another study in rice noticed only 2.8% DEGs in hybrid [58]. These studies enabled us to understand that only a small number of genetic differences can produce superior performance in hybrids.

### Cotton hybrids exhibit parent expression level dominance at squaring stage

Allopolyploids have been found to show expression level dominance (ELD), a phenomenon in which gene expression of progeny is statistically similar to that of one parent [59]. Our analysis results found most hybrid DEGs had non-additive gene expression at squaring stage especially high/low maternal or paternal like expression groups had highest portion. DEGs with additive or mid parent expressions were quite low. It is possible to assume that crossing of different genomes of two parents may cause genomic dominance of ideal parent and these changes can be a reason of better performance in hybrids. Interestingly, these hybrids had higher number of non-additive but overdominance genes expressions at seedling stage [60]. Previously, synthetic and natural allopolyploids of cotton were found to mimic the expression patterns similar to one of two diploids parent [59]. A transcriptome analysis in super hybrid rice found 30–50% non-additive gene expression at the early developmental stages [57]. Many maize scientists noticed higher non-additive gene expression [61–63], while others detected higher additive gene expression in hybrids [64, 65]. The predictions about additive genes involved in heterosis are considered because in many cases, the number of DEGs between parental lines was more than those between parental lines and F_1_ hybrids. Plenty of recent studies have implemented ELD classification to check gene expression patterns in hybrids. For instance, 70–80% of the non-additive genes in *B. napus* F_1_ hybrid displayed high parent expression level dominance during the early flowering development stage [56]. Most of hybrid DEGs in wheat followed the expression of maternal parent in seedling tissues and paternal parent in spike tissues [66]. Overdominance expressions in heterotic hybrids compared to non-heterotic hybrids have also been reported in some crop hybrids [67, 68]. Based on the results of many studies, it is still unclear which expression class is important, but it seems both additive and non-additive gene expressions probably produced heterosis in plants.

### Possible roles of signaling and sugar metabolic pathways genes in yield heterosis

Our investigation found DEGs of hybrids with P-ELD were enriched plenty of diverse pathways. However, the plant hormone signaling and sugar metabolisms were the most significance pathways. Changes in the expression of signaling genes related to Auxin response factors (ARF), Leucine-rich repeat protein, Jasmonate-zim (JAZ) domain protein, MAP kinase, and Brassinosteroid signaling regulator (BZR) proteins were found in this study. Plant hormones are a structurally distinct group of key molecules that regulate plant growth and control feedback linked with both biotic and abiotic stresses. Phytohormone signaling pathway genes linked with brassinosteroids and gibberellic acid play critical role in plant height regulation of hybrid maize [69]. Further, transcriptome comparison in *B. napus* specified that IAA and SA response genes had differential expression in F_1_ hybrids, which finally triggered hybrid vigor [56]. Auxin control transcription through IAA proteins and ARFs. IAA proteins bind with the ARF and repress transcription with the help of co-repressor called topless [70]. Transcription is either inhibited or activated depending upon the kind of an ARF which binds DNA [71]. Plant hormone signaling and responses usually make major changes in transcription. However, these are not as well categorized as the transcription responses in plants [72]. At the advent of abnormal conditions or stresses, plants generate diverse sugar and hormone signaling to maintain the balance of metabolic processes by changes at the transcript, protein, and metabolite level [73]. Earlier transcript analysis revealed sugar and auxin signaling regulate anther development during high-temperature stress in cotton [74].

To a particular interest, many sugar metabolic genes associated with Cellulose synthase (CESAs), Pectin lyase like (PEL), Sucrose synthase (Sus), Starch synthase, Alpha-beta-amylase, Pentatricopeptide repeat (PPR) and Tetratricopeptide repeat (TPR) protein had differential expression in hybrids. Cellulose and pectin are key components of plant cell walls and play role during cotton fiber cell development. Cellulose synthesis control fiber secondary wall thickening [75]. Reduced *PEL* gene transcripts caused a reduction in their enzymatic activity which ultimately reduced fiber elongation in cotton [76]. Mutants of cellulose synthase 6 (*CESA6*) showed strong cellulose deficit as a consequence short hypocotyl phenotype was witnessed in Arabidopsis [77]. A genome-wide association study in *B. napus* determined *CESA6* can be a promising candidate gene for stem lodging resistance [45]. Photosynthate in plants is transported in the form of sucrose and regulated by Sus enzymes through active participation in phloem unloading process. Sus activity associated genes perform a pivotal role in cotton fiber development by providing sucrose and cellulose to growing fiber [78, 79]. Additionally, Sus activity has strong connection with the final harvest index of starch storing organs in the plant. Starch is a carbon storage polymer and acts as a major regulator of plant growth through balancing net carbon availability. The carbon fixed by plants is stored as starch in the light which is completely utilized in the night for respiration and growth [80]. Thus, any change that leads to a temporary state of carbon shortage, reduce the rate of plant growth by rebalancing the net carbon [81, 82] through enhancing the rate of starch synthesis and reducing the rate of starch breakdown [83, 84]. Gene expression study in hybrid rice has shown that several genes linked with circadian rhythm, carbon fixation, and starch and sucrose metabolism were located into yield QTLs regions, expecting these putative candidate genes for yield heterosis [85]. Besides photosynthesis, other metabolic pathways such as sucrose and starch may be a key contributor to wheat heterosis [86]. Maize seed and potato tuber contain plenty of starch on dry weight basis, so their yield mainly depends on starch and sucrose metabolism [87, 88]. Starchless mutants cannot grow in a light/dark cycle due to an imbalance of carbon and starch which ultimately produced carbon shortage at night resulting in growth impedance for quite a few hours the next day [81].

### Role of QTLs and co-expression network to excavate candidate genes for yield heterosis

Quantitative trait locus (QTLs) provides an association between genomics and phenomics of complex traits. In the last decade, several researchers have performed QTLs analysis to understand heterosis [42]. However, difficult to point out target genes in most cases due to the large region of the identified QTLs. To minimize this challenge, a promising approach is mapping DEGs on already known QTLs and then the construction of their co-expression regulatory network. Further, yield heterosis is a reflection of the effect of many interconnected genes rather than individual genes, thus understanding the interrelationship between genes facilitates to exploit candidate genes that are likely associated with a biological system. A similar approach already has been done in rice and *B. napus* [45, 58]. Our selected hybrids were superior or inferior to their parents in seed cotton yield, and investigation found 74 DEGs from key pathways were mapped on 43 overlapped seed cotton yield QTLs.

Our integrated co-expression network analysis of high and low hybrids using green module revealed nine seed cotton yield QTLs mapped genes, interconnected with many genes enriched in biological and molecular functions such as carbohydrate metabolic processes, catalytic activities, and protein binding or transportation. Our identified putative candidate genes were *Gh_A03G1024*, *Gh_D08G1440, Gh_A08G2210, Gh_A12G2183, Gh_D07G1312, Gh_D08G1467, Gh_A03G0889, Gh_A08G2199,* and *Gh_D05G0202.* More interestingly, all these genes showed tissue-specific differential expression in reproductive tissue flower buds and 1 DPA ovule of hybrids. Gene *BZR1* is associated with brassinosteroid mediated signaling pathway and regulates cell elongation and division. *BES1* transgenic plants in upland cotton showed reduced hypocotyl sensitivity to brassinazole, curled leaves, bent petioles, and changed expression of BR constitutive genes [89]. OsBRI1 gene functions in various growth and developmental processes in rice, caused dwarfism, and bending of the lamina [90]. *ASK8* is a Shaggy-related gene that enables extracellular signals to adjust transcription in differentiating cells, stress responses [91], and also participate in biological functions linked with BR response. The gene *Gh_A08G2210* belongs to endoglucanase gene family and involved in cell wall organization and cellulose catabolism. It was predicted that it may have a function in pollen and pollen tube growth in Arabidopsis [92].

Other putative candidate genes associated with starch biosynthesis such as *GBSS1* and *APL2* showed differential expression in flower buds of high hybrid. CRISPR/Cas9 mediated mutations of *GBSSI* effect reproductive growth as a result shrunken seeds were produced in rice [93]. Additionally, promising candidate signaling genes associated with *JAZ10* and *MPK4* protein showed differential expression in only 1 DPA ovule of low hybrid. *MPK4* modifies the expression of genes responding to biotic and abiotic stresses, and has an important role in pathogen defense [94]. More to this, it also take part in regulation of cytokine [95], salicylic acid, and jasmonic acid-mediated defense gene expression. *JAZ10* gene acts as repressor of jasmonate responses and Jasmonoyl isoleucine specifically promotes *COI1-TIFY10A/JAZ1* interaction as observed in Arabidopsis [96]. Gene *PHO1* thought to be involved in the transportation of inorganic phosphate [97], here in our results showed downregulation in 1 DPA ovule of low hybrid. Chloroplast *CRR21* gene play the main role in chloroplast RNA editing and modifications [98]. Interestingly, this gene showed downregulation in fiber ovule of low hybrid only. To be concise, the integration of transcriptomic, QTLs and co-expression network provide useful understanding about the genomics of seed cotton yield heterosis.

## Conclusions

Notably, F_1_ hybrids of upland showed meaningful mid and better heterosis in seed cotton yield. Comparative transcriptome study of contesting hybrids and their inbred parents at squaring stage revealed many DEGs with parent like gene expressions. In addition, many auxins, brassinosteroid, jasmonic acid hormone signaling and cellulose, sucrose, and starch synthase metabolic DEGs were mapped on known seed cotton yield QTLs. Co-expression network analysis identified nine promising candidate genes and discovered their complex biological network with interconnected genes that probably mediate seed cotton yield heterosis. Together all, our field experimentation quantified hybridization in cotton is useful to improve yield. Comprehensive genome-wide transcriptome analysis gave new insights to understand the preliminary genetics of yield heterosis. However, further research at the gene functional level is desired to understand the perturbed biological system of yield heterosis in upland cotton.

## Methods

### Calculation of phenotypic heterosis for yield parameter

In 2015, our research group produced 30 intraspecific F_1_ upland cotton hybrids by using 11 inbred parents. The brief detail about all plant material and two-year field experimentation at three locations can be seen in our previous study [99]. The phenotypic data from all field experimentation were used to determine the degree of heterosis in yield traits, as mid parent heterosis (MPH), was calculated using the equation: MPH = 100 × (F_1_ - MP)/MP, and that of better parent heterosis (BPH) was calculated using the equation: BPH = 100 × (F_1_ - BP)/BP, where F_1_ is the performance value of each trait for the F_1_ progeny, MP is the mean value of that trait for the parents, and BP is the value of that trait for the better parent.

### RNA extraction, Illumina sequencing, and data analysis

Based on the level of phenotypic heterosis in yield traits, high (denoted as H), medium (M), and low (L) hybrid and their four inbred parents were selected for transcriptomic analysis at squaring stage which is generally the start of reproductive stage in cotton. Inbred lines used in this study were self-fertilized for many generations to maintain purity and included one maternal inbred line named SJ48 (denoted as A) and three paternal inbred lines viz. Z98–15 (B), 851–2 (C), and DT-8 (D). The detail about field experimentation is described in our published study [60]. For RNA samples, leaf, flower buds, and 1 day after post anthesis (1 DPA) ovule in three biological replicates were collected for all seven materials from the field test of the year 2018. All plant materials and field localities used in this study were the property of Institute of Cotton Research of Chinese Academy of Agricultural Sciences, Anyang, China. Samples for leaf and flower buds were picked at squaring stage. At the same time, fully opened flowers from random plants were tagged to pick 1 DPA ovule. Young leaves about 2 mg picked from different plants were pooled for the composite samples. Similarly, flower buds of 2 mm, 3 mm and 4 mm size were also pooled. In all, a total of 63 samples were used to isolate total RNA. After initial quality measurements and preparations, paired-end sequencing (300 ± 50 bp) on an Illumina Hiseq 4000 was implemented at the LC biosciences China by following the vendor’s recommended protocol. Cutadapt 1.10 [100] and Perl scripts in house were used for quality control. HISAT 2.0 [101] was applied to aligned clean reads to the upland cotton genome [36]. StringTie 1.3 [102] was used to assemble mapped reads. After the final transcriptome was generated, StringTie 1.3 together with Ballgown [103] was applied to estimate the expression levels. The expression profiles for mRNAs in the FPKM form were detected with StringTie 1.3. The criteria log2 (fold change) > 1 or < − 1 and *p-value < 0.05* was used to identify differentially expressed mRNAs between two samples. The analysis of expression level dominance was executed with density estimator in the R software package by opting the method as previously described [59]. By this analysis, expression statistics among two parents and their derived hybrids were distributed into following 12 possible groups. Genes expression in hybrids may be additive (1 and 2 groups), paternal-expression level dominance (3 and 4 groups), maternal-expression level dominance (5 and 6 groups), and transgressive expression lower or higher than either parent (7 to 12 groups). The GO functional annotations of DEGs were tested with the Goatools software [104]. KOBAS software [105] was used to retrieve the KEGG pathway enrichment analysis. The threshold criteria *p-value ≤ 0.05* and rich factor was applied for enrichment analysis.

### Mapping key DEGs on seed cotton yield QTLs

Seed cotton yield QTLs (LOD > 2) with genetic position and linked molecular markers were acquired from the cotton QTL database (www.cottonqtldb.org). The physical positions of QTLs, gene loci, and coordinates were attained from the upland cotton genome [36]. Depend on the physical positions of both gene loci and QTL. DEGs from highly enriched and key pathways were then mapped to QTLs. Mapchart software 2.0 was used to display the physical location of genes and QTLs on concerned chromosomes.

### Weighted gene co-expression network analysis

WGCNA was performed individually in high and low hybrids by using squaring stage DEGs. These DEGs were identified in comparative RNA-seq data analysis between these hybrids and their respective parents and pool from leaf, flower buds, and 1 DPA ovule tissues using each biological replicate as an individual dataset. Biologically appropriate network was constructed by following step by step method as previously described [106]. In WGCNA, modules dendrograms were made by using dynamic tree cut method. While modules were identified on the basis of merge CutHeight method. Different genes were clustered into different modules due to their correction weight and expression profile. Finally, we selected modules having the highest connectivity from each hybrid to construct a network. The co-expression network was built using only putative candidate genes (Genes enriched both in seed cotton yield QTLs and key pathways) and their interconnected genes having weights > 0.40. Cytoscape_v 3.7.1 software [107] was used to show final co-expression network.

### qRT-PCR analysis

For qRT-PCR, same total RNA prepared for RNA sequencing was used to synthesis first standard cDNA fragments for each sample with PrimerScript™ RT Reagent Kit for Perfect Real Time (RR037A, TaKaRa, Japan). Oligo7 software was used to design gene-specific primers, and reaction mixture for qRT-PCR was prepared using TransSart Top Green qPCR SuperMix (AQ131,TransGen Biotech, China). The actin gene was used for normalization. Three biological replicate each with three technical replicates were run for target and actin genes. The protocol of qRT-PCR and data analysis technique were same as described in our previous study [17].

## Supplementary information

**Additional file 1: Figure S1.** The mapped region’s statistics of all 63 sequenced libraries of parents and hybrids. Here, RL: leaf, F: flower buds, DPA: 1 day post anthesis ovule, A: maternal parent, and B, C, D represents three paternal parents of high (H), medium (M), and low (L) hybrids, respectively. Numerical values 1, 2, 3 correspond to three biological replicates.

**Additional file 2: Figure S2.** Principle component analysis for all samples. In the figure, RL: leaf, F: flower buds, DPA: 1 day post anthesis ovule, A: maternal parent, and B, C, D represents three paternal parents of high (H), medium (M), and low (L) hybrids, respectively. R1, R2, R3 correspond to three biological replicates.

**Additional file 3: Figure S3.** Pearson correlation between different samples. Here, RL: leaf, F: flower buds, DPA: 1 day post anthesis ovule, A: maternal parent, and B, C, D represents three paternal parents of high (H), medium (M), and low (L) hybrids, respectively. Numerical values 1, 2, 3 correspond to three biological replicates.

**Additional file 4: Figure S4.** Total number of expressed genes for each sample. In this figure, RL: leaf, F: flower buds, DPA: 1 day post anthesis ovule, A: maternal parent, and B, C, D represents three paternal parents of high (H), medium (M), and low (L) hybrids, respectively. Numerical values 1, 2, 3 correspond to three biological replicates.

**Additional file 5: Figure S5.** Expression heatmap of parent like expressed genes of high hybrid. In each figure, RL: leaf, F: flower buds, DPA: 1 day post anthesis ovule, A: maternal parent, and B: paternal parent, H: high hybrid, respectively. Numerical values 1, 2, 3 correspond to three biological replicates.

**Additional file 6: Figure S6.** Expression heatmap of parent like expressed genes of medium hybrid. In each figure, RL: leaf, F: flower buds, DPA: 1 day post anthesis ovule, A: maternal parent, and C: paternal parent, M: medium hybrid, respectively. Numerical values 1, 2, 3 correspond to three biological replicates.

**Additional file 7: Figure S7.** Expression heatmap of parent like expressed genes of low hybrid. In each figure, RL: leaf, F: flower buds, DPA: 1 day post anthesis ovule, A: maternal parent, and D: paternal parent, L: low hybrid, respectively. Numerical values 1, 2, 3 correspond to three biological replicates.

**Additional file 8: Figure S8.** Most enriched GO terms for DEGs with parents like expression in hybrids at squaring. **a, b,** and **c** shows GO terms with total number of genes in high (H), medium (M) and low (L) hybrid, respectively. Here, most enriched GO terms with *p* < 0.05 are only presented in each figure.

**Additional file 9: Figure S9.** Common GO and pathways of parent like differentially expressed genes of hybrids. **a** and **b** respectively represents GO and pathways among high (H), medium (M) and low (L) hybrids.

**Additional file 10: Figure S10.** Expression heatmap of known seed cotton yield QTLs mapped genes of hybrids. **a** shows high hybrid parent triad specific gene in leaf (RL), flower buds (F), and 1 DPA ovule. **b** represents low hybrid parent triad specific genes. Here, A, B, and D represent inbred parents and H, and L correspond to high and low hybrids.

**Additional file 11: Figure S11.** Modules dendrogram, correlation, and average connectivity high and low hybrids. **a** and **b** represents cluster dendrograms showing co-expressed modules in high (H) and low (L) hybrids respectively. **c** shows modules correlation between a: high and b: low hybrid. **d** indicates the average connectivity of genes in each module of high and low hybrid.

**Additional file 12: Figure S12.** Expression heatmap of nine key genes of hybrids. **a** shows low hybrid specific expressed four genes. **b** indicates high hybrid specific expressed five genes. Here, F: flower buds, DPA: 1 day post anthesis ovule, A: maternal parent, B, and D represent paternal parents of high (H) and low (L) hybrids.

**Additional file 13: Figure S13.** qRT-PCR of nine selected putative candidate genes. In each figure, H: High, M: Medium, L: Low and A, B, C, and D stands for four inbred parents. The first five genes show expression in flower buds tissue and others represent expression in 1 DPA ovule. * shows a significant difference in hybrids only with their one parent and ** with both parents at *p-value 0.05*.

**Additional file 14: Table S1.** Mean of mid and better parent heterosis observed for yield traits in three locations and two-year field experimentation.

**Additional file 15: Table S2.** Summary of RNA sequencing and mapping for all 63 samples.

**Additional file 16: Table S3.** Most enriched GO terms for parent like expressed DEGs of high, medium, and low hybrids.

**Additional file 17: Table S4.** Most enriched pathways for parent like expressed DEGs of high, medium, and low hybrids.

**Additional file 18: Table S5.** Detailed information of already reported seed cotton yield QTLs used to map DEGs.

**Additional file 19: Table S6.** Number of co-expressed modules and genes for hybrids.

**Additional file 20: Table S7.** List and weightage of co-expressed SCY-QTLs mapped genes from blue module.

**Additional file 21: Table S8.** List of gene primers used for qRT-PCR.

## Data Availability

All sequencing datasets generated or analyzed in this study are freely available with accession number GSE150052 at https://www.ncbi.nlm.nih.gov/geo/query/acc.cgi?acc=GSE150052.

## Abbreviations

DEGs: Differentially expressed genes
MPH: Mid parent heterosis
P-ELD: Parent expression level dominance
QTLs: Quantitative trait locus
WGCNA: Weighted gene co-expression network analysis
