# Supplementary figures and images for: Comparative transcriptome analysis between inbred and hybrids reveals molecular insights into yield heterosis of upland cotton
Source: BMC Plant Biol. 2020 May 27;20:239. doi: 10.1186/s12870-020-02442-z (PMC7251818; doi:10.1186/s12870-020-02442-z)

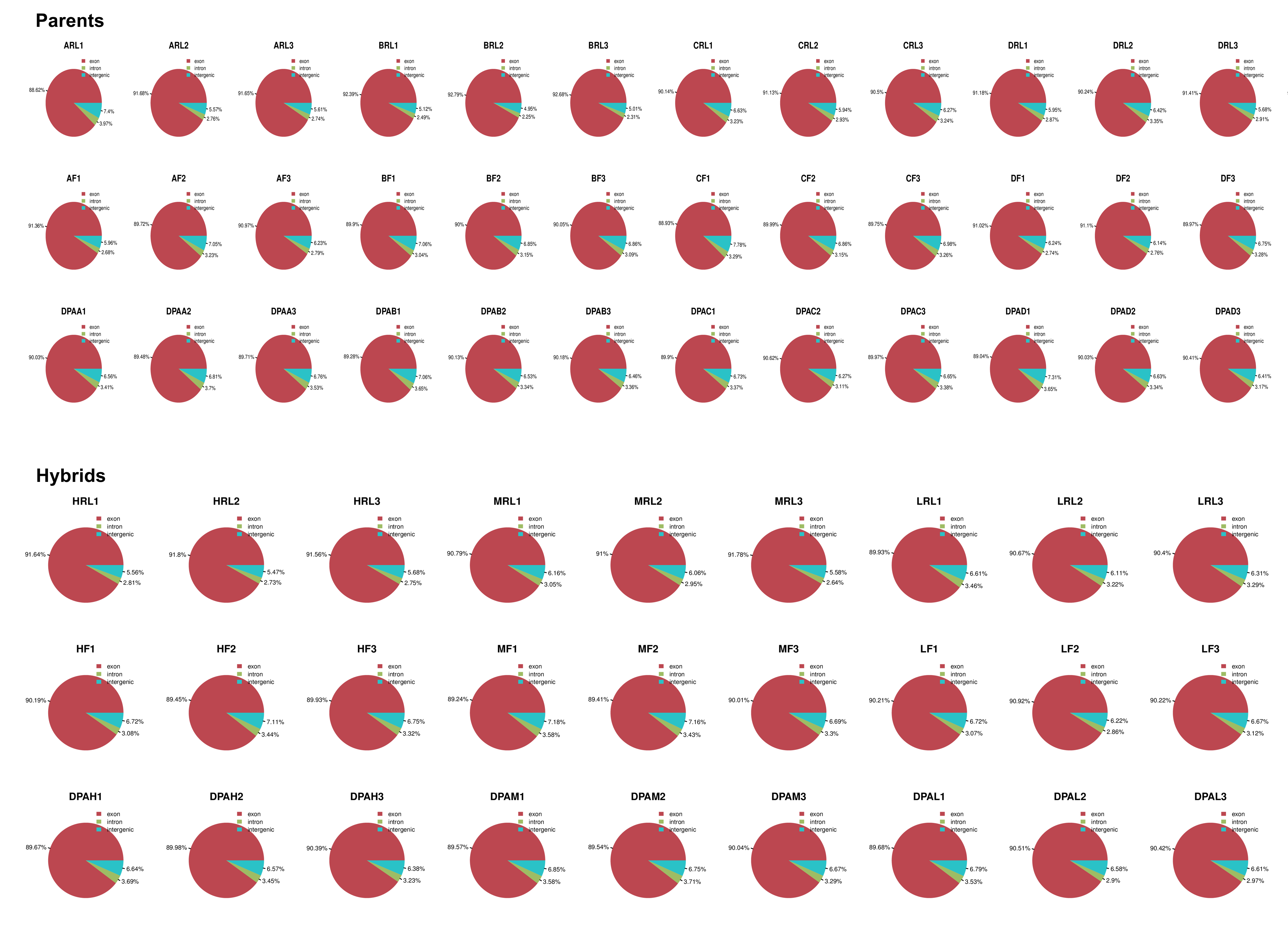

Supplement: Supplementary file 1 — Additional file 1: Figure S1. The mapped region’s statistics of all 63 sequenced libraries of parents and hybrids. Here, RL: leaf, F: flower buds, DPA: 1 day post anthesis ovule, A: maternal parent, and B, C, D represents three paternal parents of high (H), medium (M), and low (L) hybrids, respectively. Numerical values 1, 2, 3 correspond to three biological replicates. [file 12870_2020_2442_MOESM1_ESM.png]

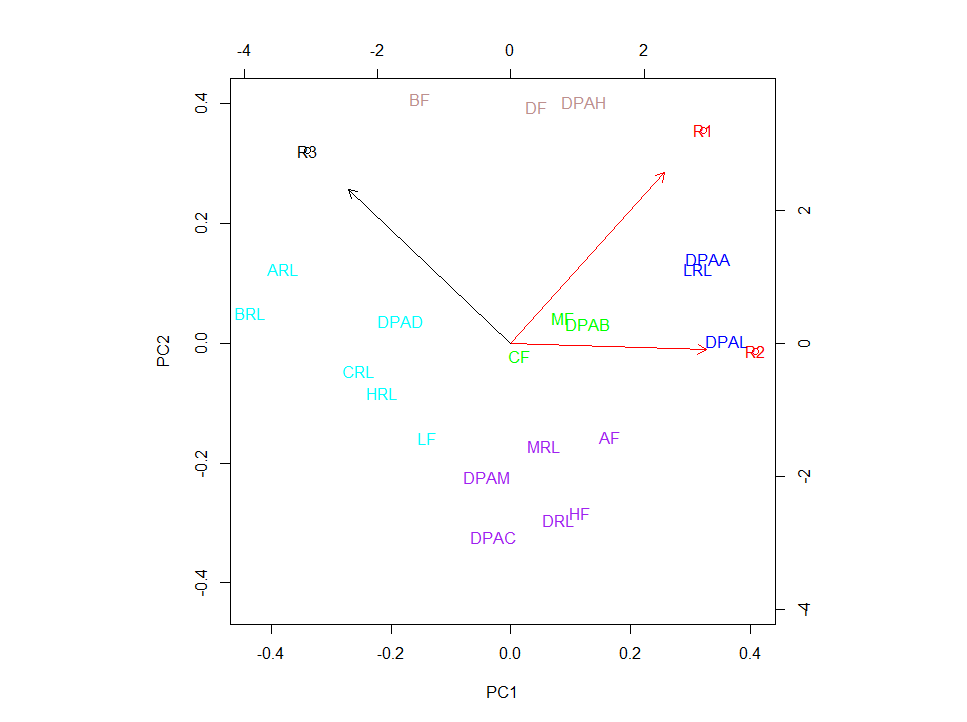

Supplement: Supplementary file 2 — Additional file 2: Figure S2. Principle component analysis for all samples. In the figure, RL: leaf, F: flower buds, DPA: 1 day post anthesis ovule, A: maternal parent, and B, C, D represents three paternal parents of high (H), medium (M), and low (L) hybrids, respectively. R1, R2, R3 correspond to three biological replicates. [file 12870_2020_2442_MOESM2_ESM.png]

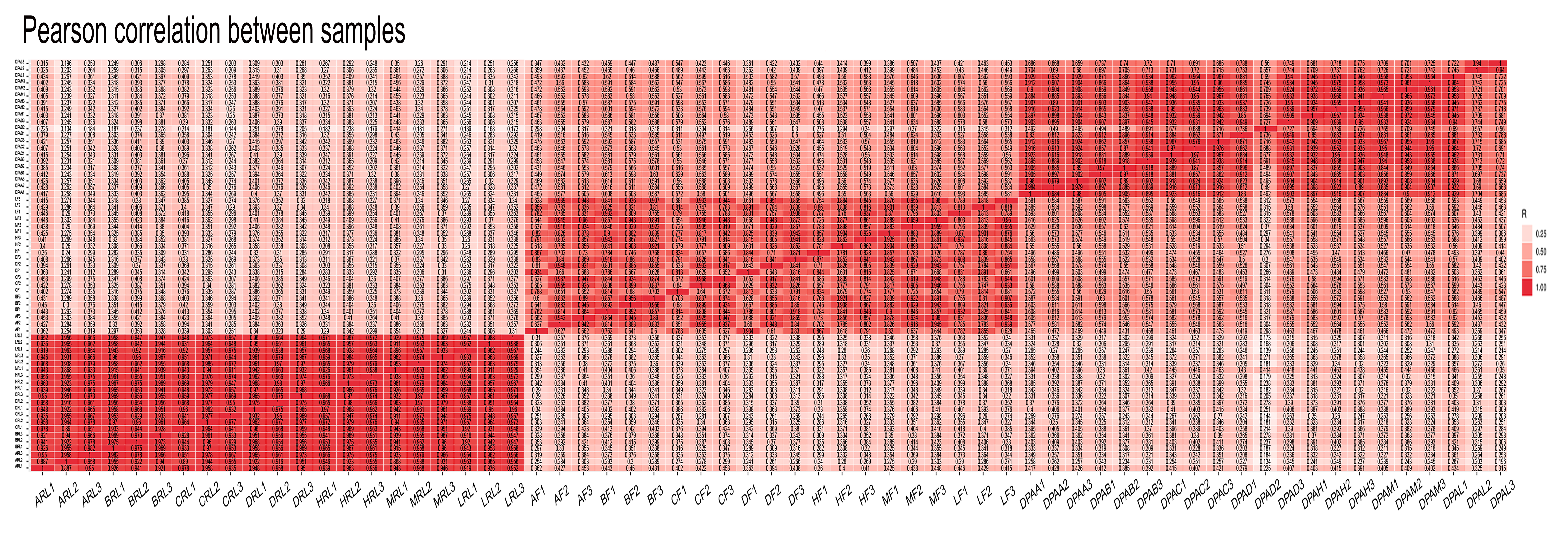

Supplement: Supplementary file 3 — Additional file 3: Figure S3. Pearson correlation between different samples. Here, RL: leaf, F: flower buds, DPA: 1 day post anthesis ovule, A: maternal parent, and B, C, D represents three paternal parents of high (H), medium (M), and low (L) hybrids, respectively. Numerical values 1, 2, 3 correspond to three biological replicates. [file 12870_2020_2442_MOESM3_ESM.png]

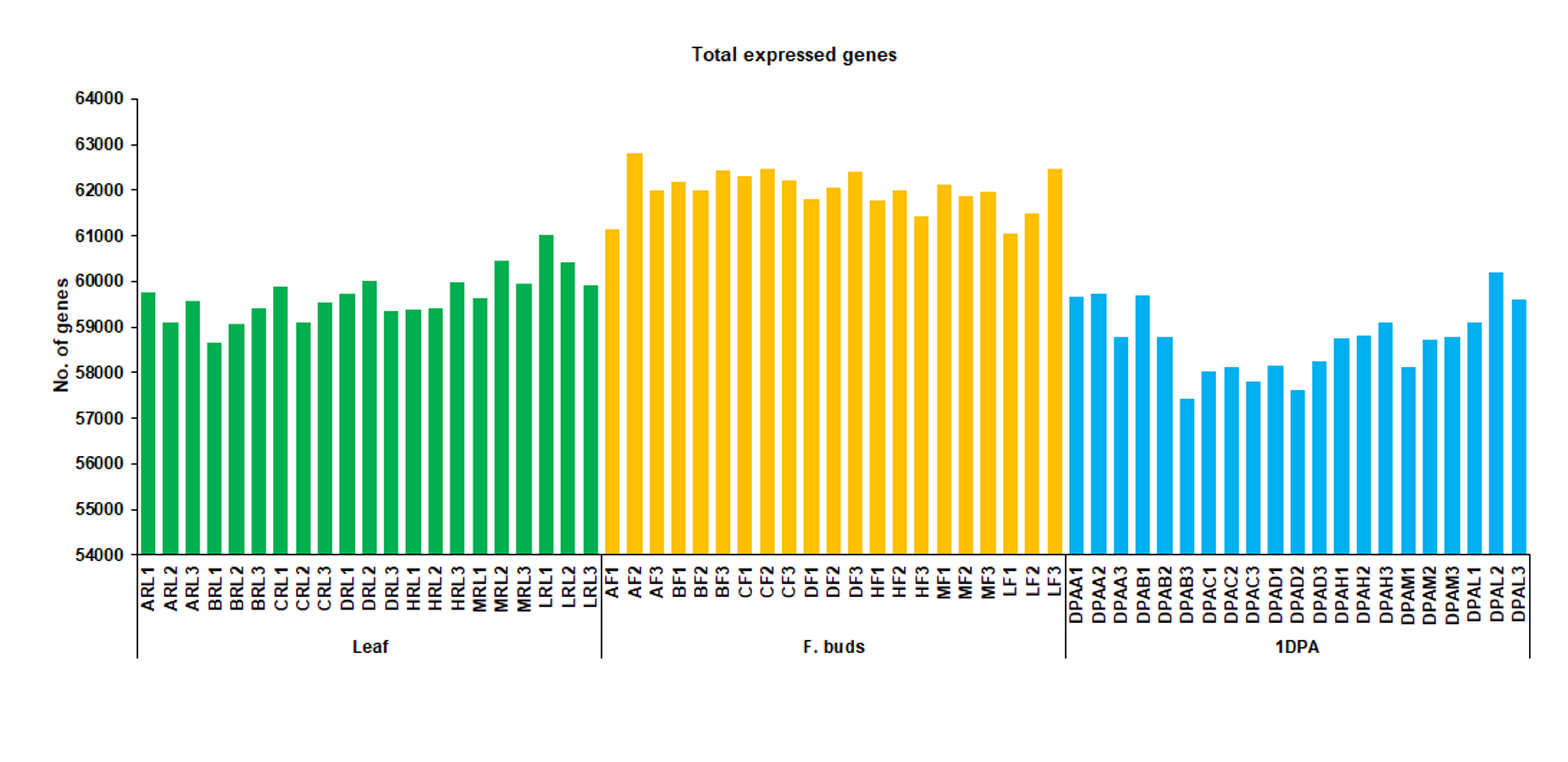

Supplement: Supplementary file 4 — Additional file 4: Figure S4. Total number of expressed genes for each sample. In this figure, RL: leaf, F: flower buds, DPA: 1 day post anthesis ovule, A: maternal parent, and B, C, D represents three paternal parents of high (H), medium (M), and low (L) hybrids, respectively. Numerical values 1, 2, 3 correspond to three biological replicates. [file 12870_2020_2442_MOESM4_ESM.png]

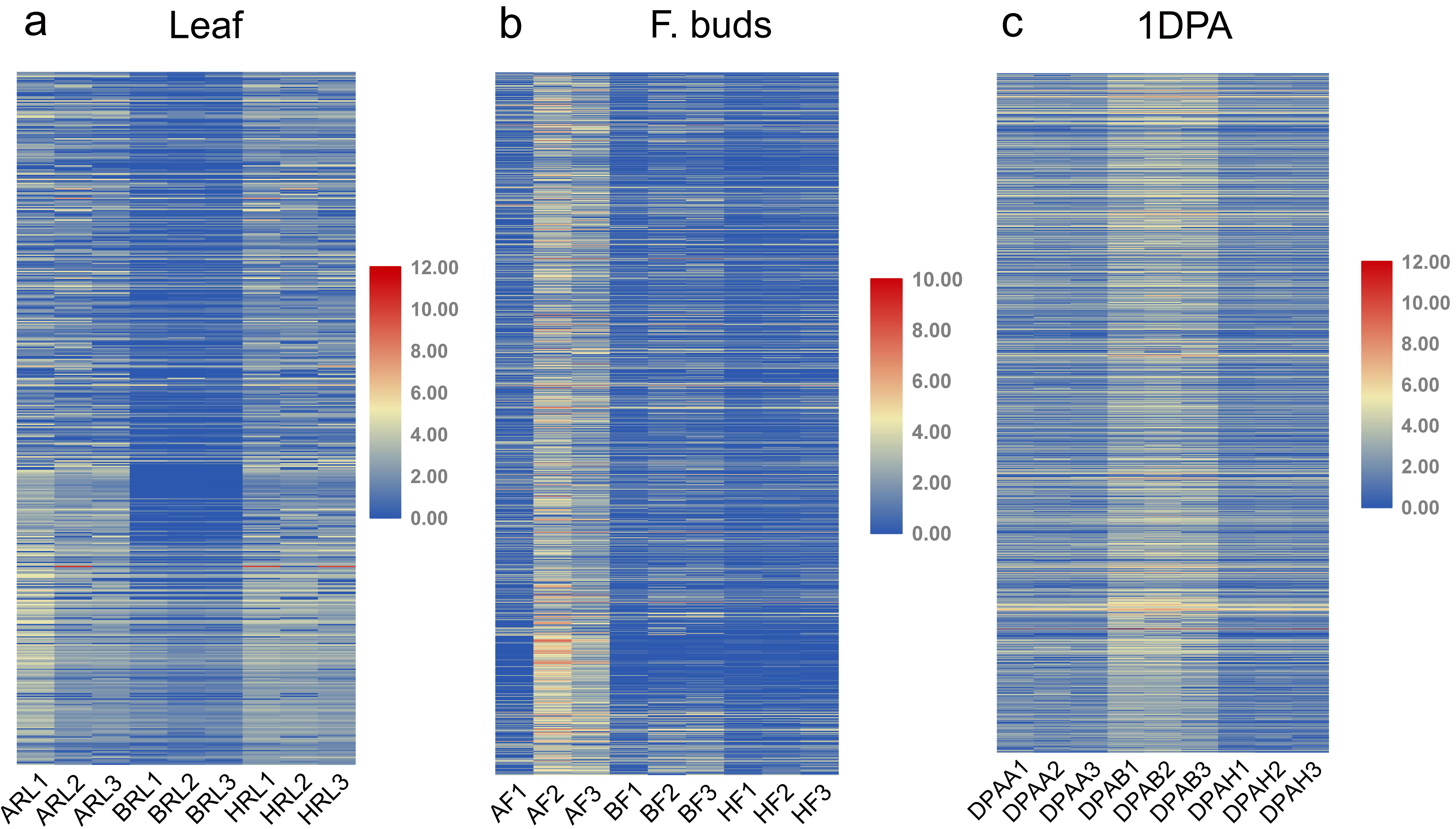

Supplement: Supplementary file 5 — Additional file 5: Figure S5. Expression heatmap of parent like expressed genes of high hybrid. In each figure, RL: leaf, F: flower buds, DPA: 1 day post anthesis ovule, A: maternal parent, and B: paternal parent, H: high hybrid, respectively. Numerical values 1, 2, 3 correspond to three biological replicates. [file 12870_2020_2442_MOESM5_ESM.png]

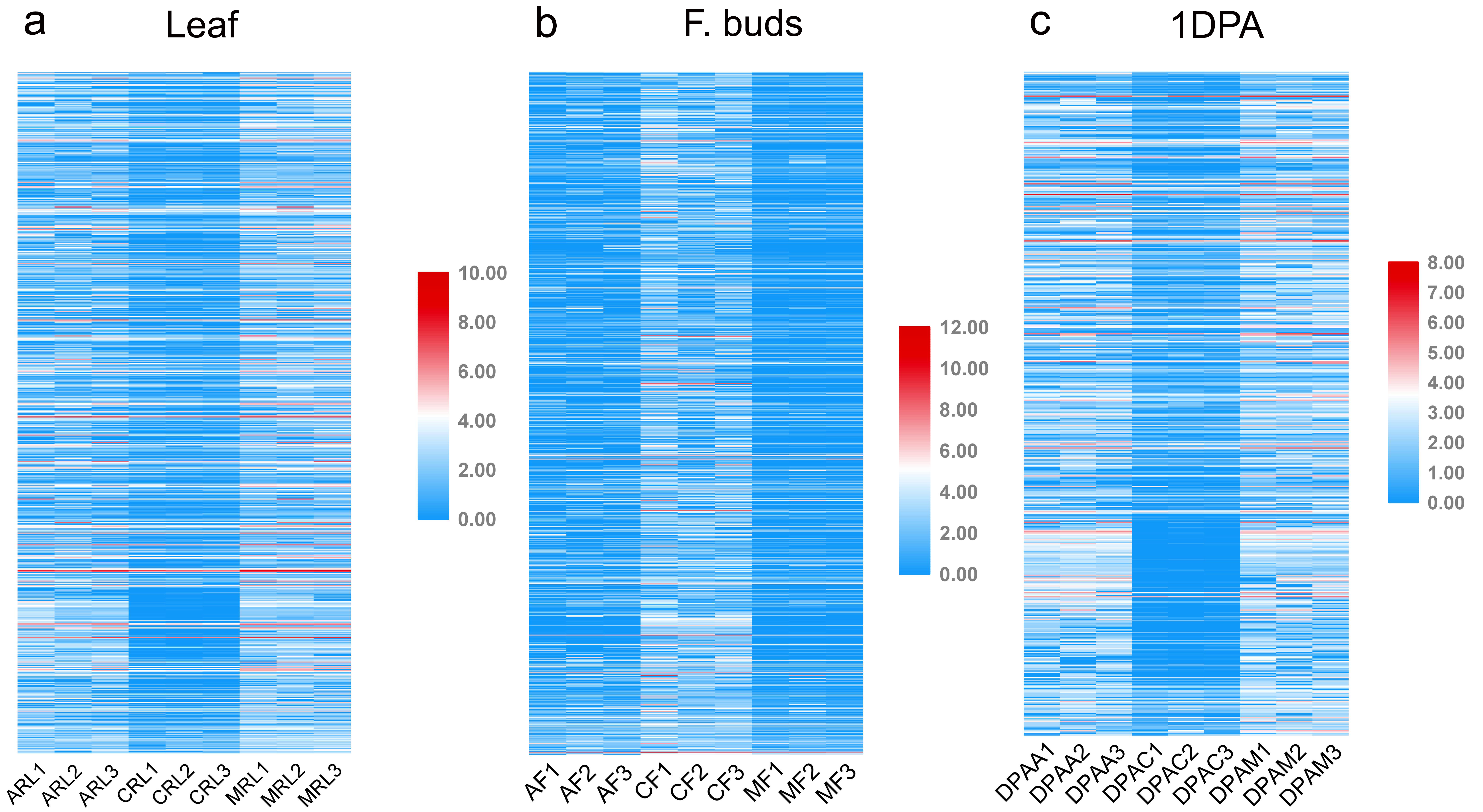

Supplement: Supplementary file 6 — Additional file 6: Figure S6. Expression heatmap of parent like expressed genes of medium hybrid. In each figure, RL: leaf, F: flower buds, DPA: 1 day post anthesis ovule, A: maternal parent, and C: paternal parent, M: medium hybrid, respectively. Numerical values 1, 2, 3 correspond to three biological replicates. [file 12870_2020_2442_MOESM6_ESM.png]

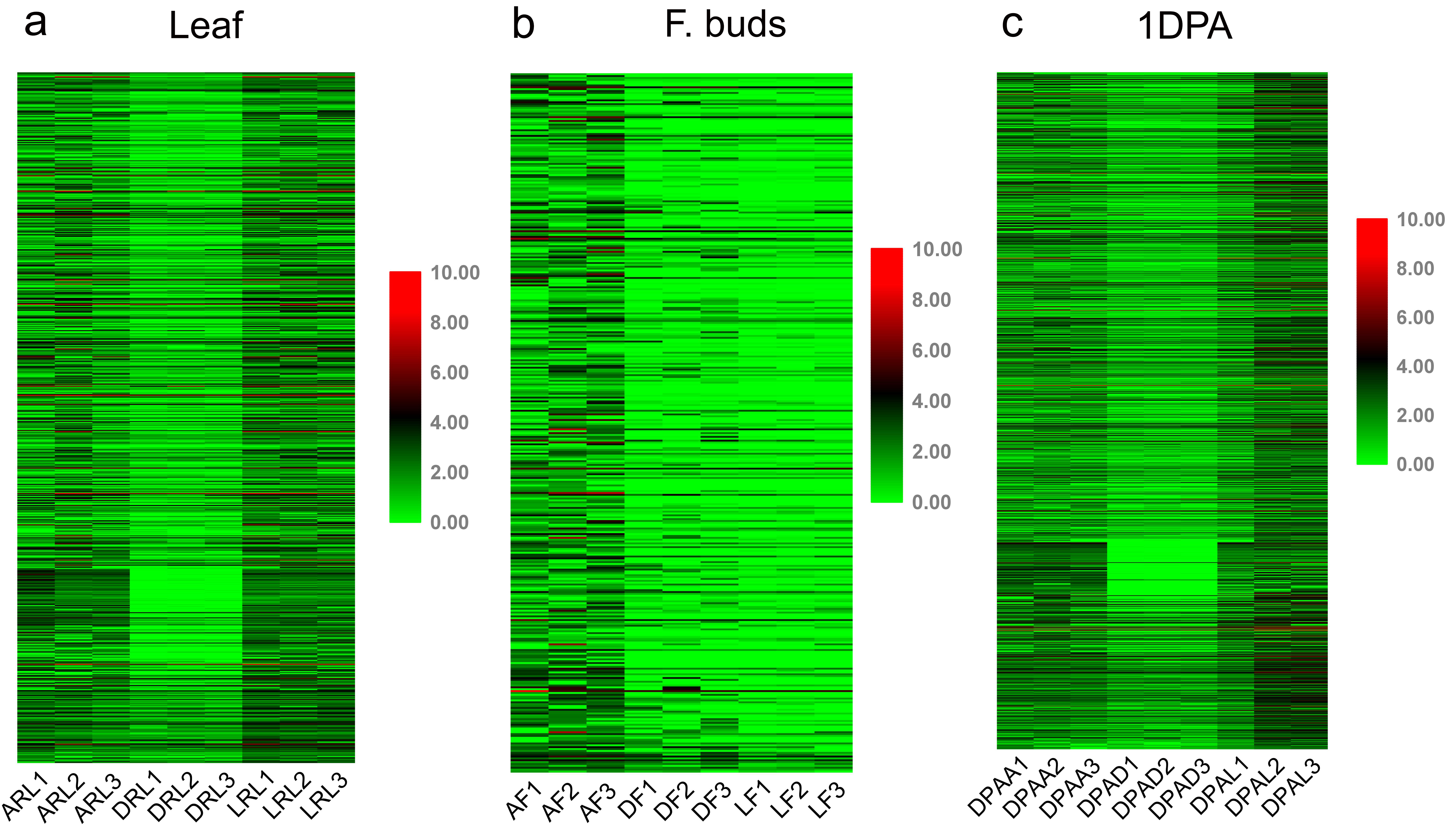

Supplement: Supplementary file 7 — Additional file 7: Figure S7. Expression heatmap of parent like expressed genes of low hybrid. In each figure, RL: leaf, F: flower buds, DPA: 1 day post anthesis ovule, A: maternal parent, and D: paternal parent, L: low hybrid, respectively. Numerical values 1, 2, 3 correspond to three biological replicates. [file 12870_2020_2442_MOESM7_ESM.png]

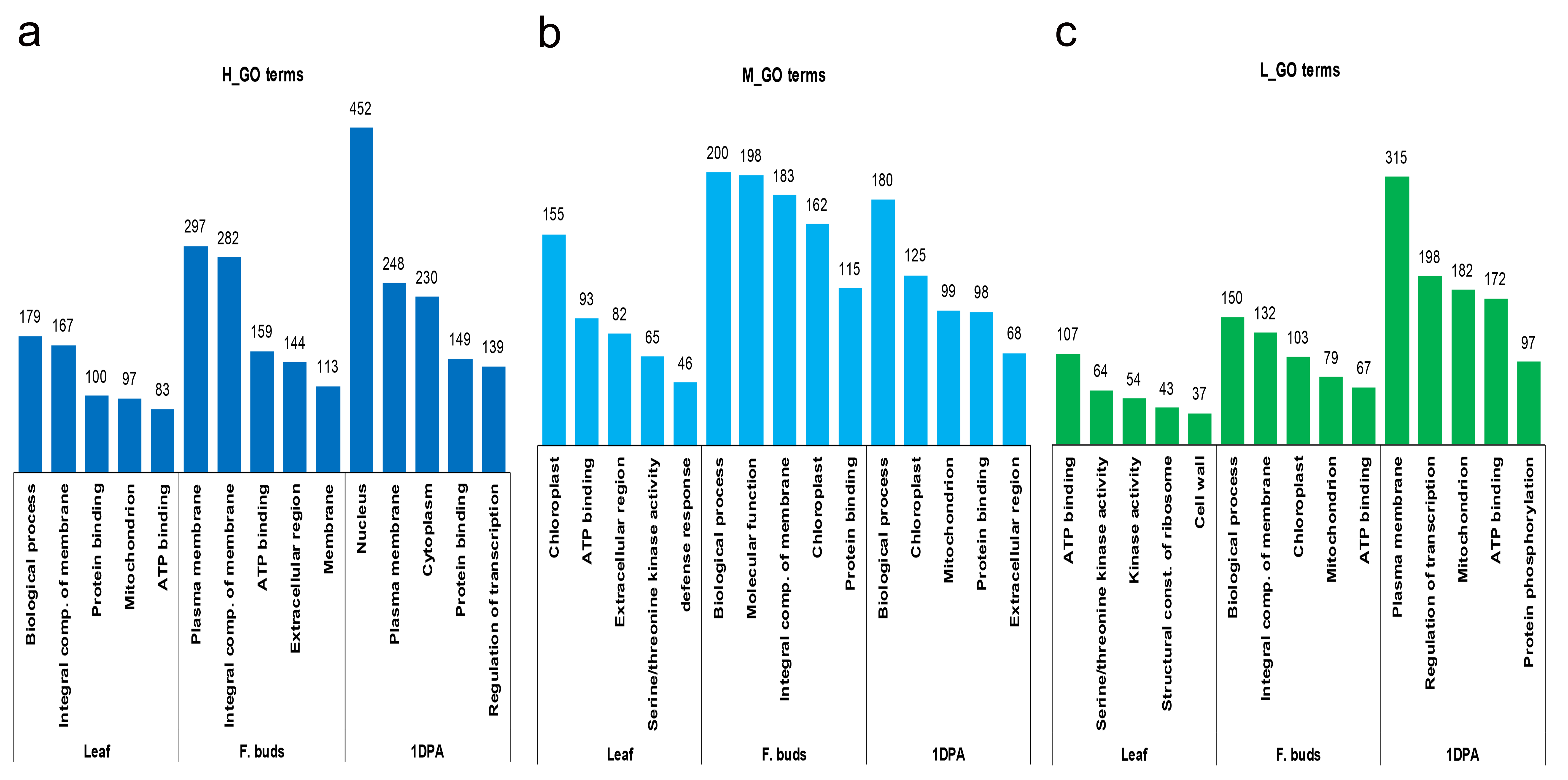

Supplement: Supplementary file 8 — Additional file 8: Figure S8. Most enriched GO terms for DEGs with parents like expression in hybrids at squaring. a, b, and c shows GO terms with total number of genes in high (H), medium (M) and low (L) hybrid, respectively. Here, most enriched GO terms with p < 0.05 are only presented in each figure. [file 12870_2020_2442_MOESM8_ESM.png]

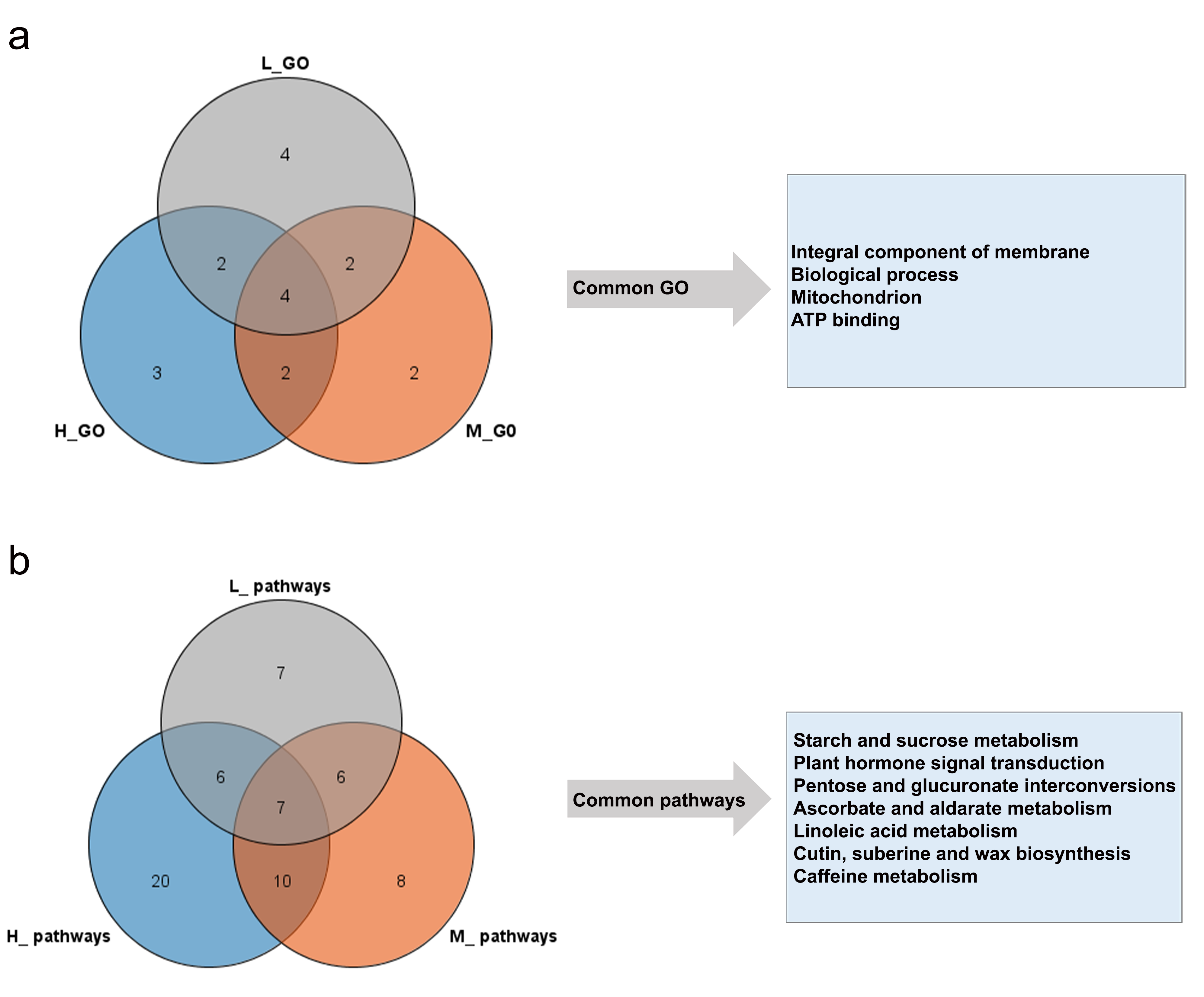

Supplement: Supplementary file 9 — Additional file 9: Figure S9. Common GO and pathways of parent like differentially expressed genes of hybrids. a and b respectively represents GO and pathways among high (H), medium (M) and low (L) hybrids. [file 12870_2020_2442_MOESM9_ESM.png]

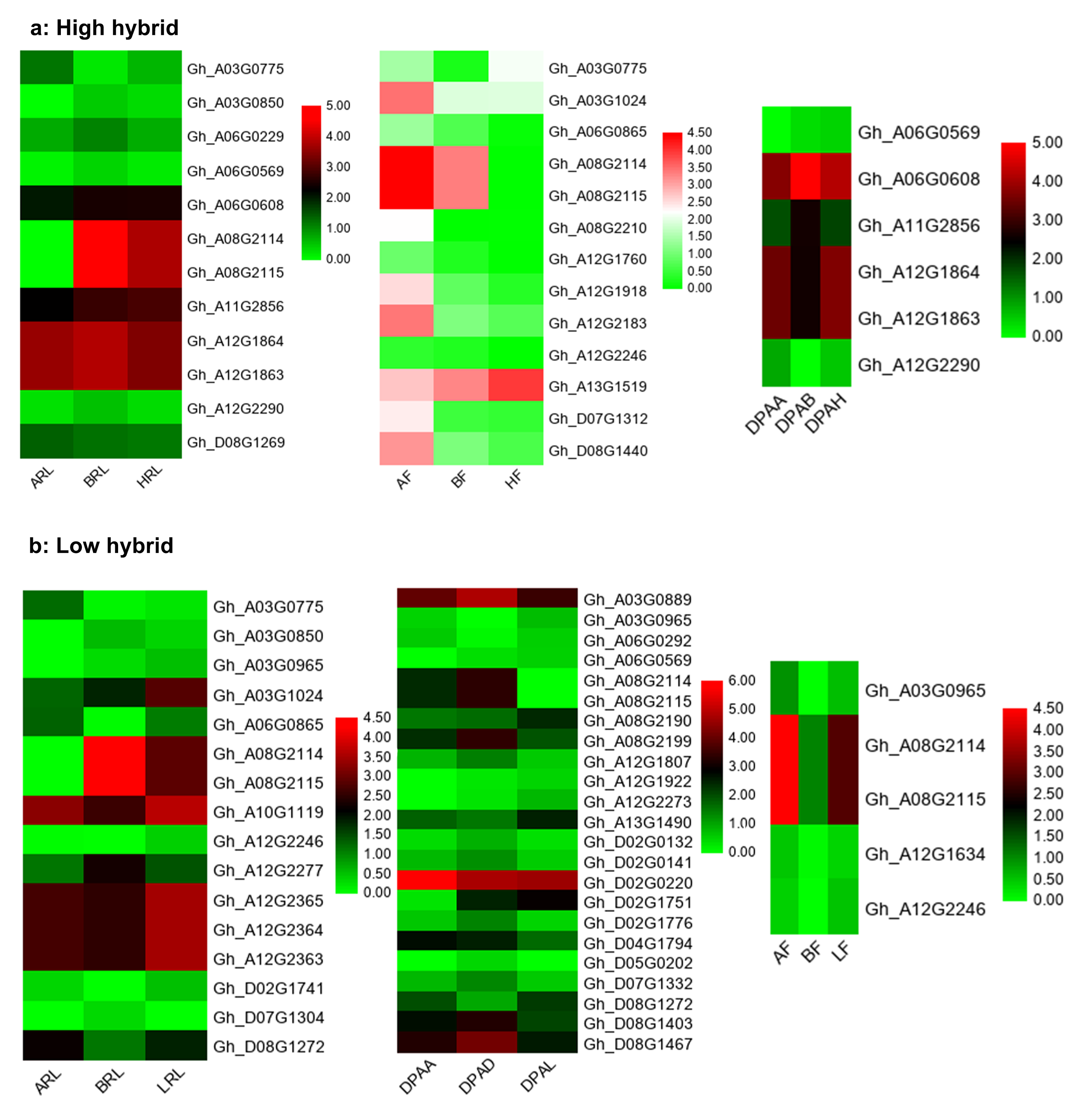

Supplement: Supplementary file 10 — Additional file 10: Figure S10. Expression heatmap of known seed cotton yield QTLs mapped genes of hybrids. a shows high hybrid parent triad specific gene in leaf (RL), flower buds (F), and 1 DPA ovule. b represents low hybrid parent triad specific genes. Here, A, B, and D represent inbred parents and H, and L correspond to high and low hybrids. [file 12870_2020_2442_MOESM10_ESM.png]

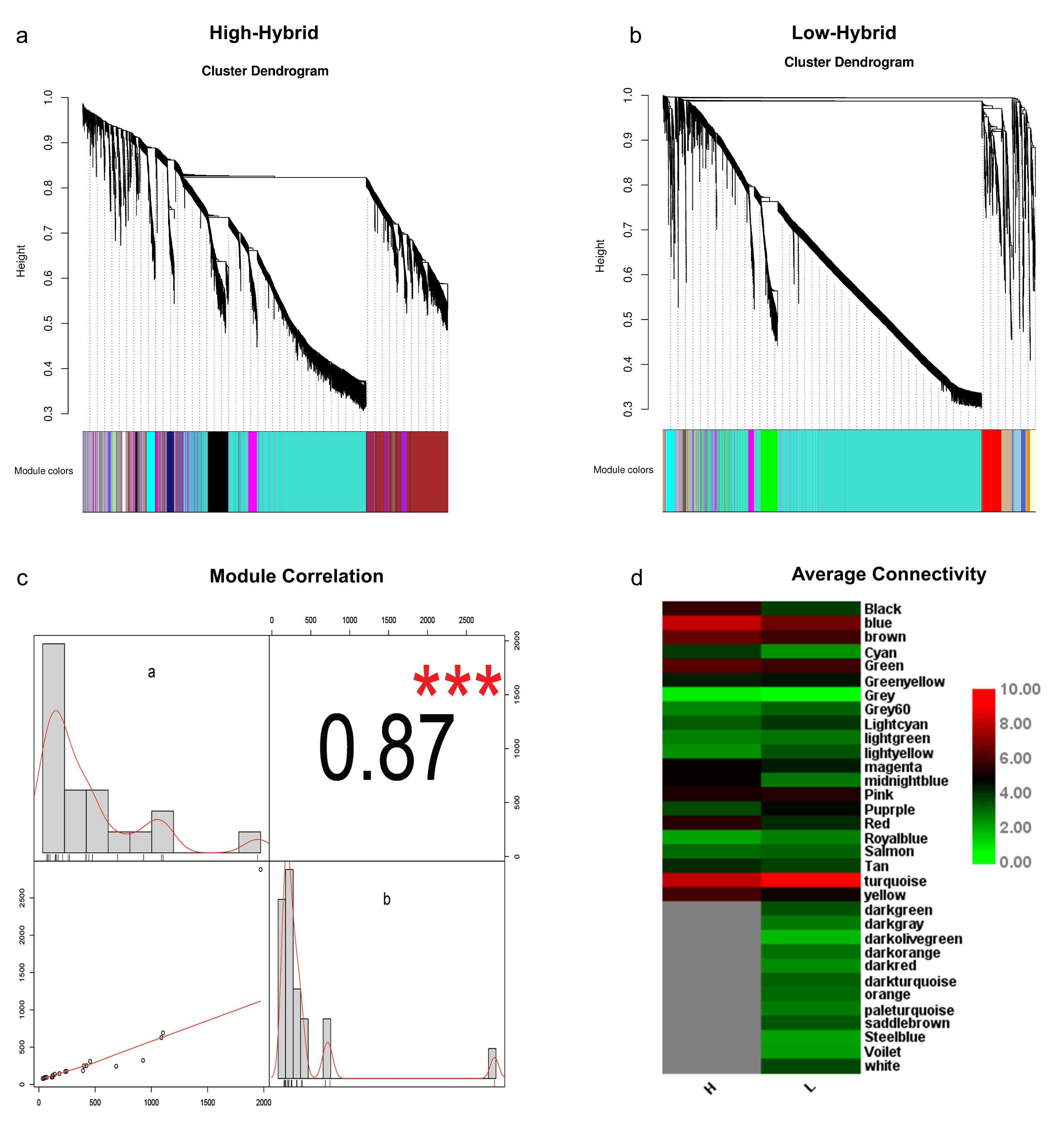

Supplement: Supplementary file 11 — Additional file 11: Figure S11. Modules dendrogram, correlation, and average connectivity high and low hybrids. a and b represents cluster dendrograms showing co-expressed modules in high (H) and low (L) hybrids respectively. c shows modules correlation between a: high and b: low hybrid. d indicates the average connectivity of genes in each module of high and low hybrid. [file 12870_2020_2442_MOESM11_ESM.png]

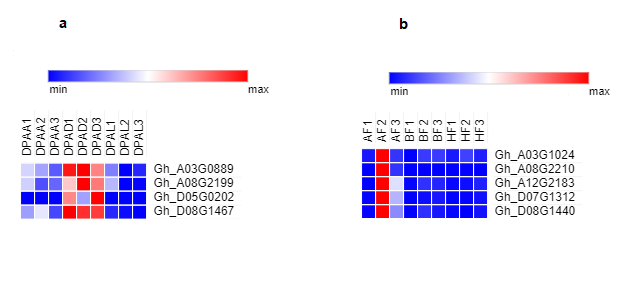

Supplement: Supplementary file 12 — Additional file 12: Figure S12. Expression heatmap of nine key genes of hybrids. a shows low hybrid specific expressed four genes. b indicates high hybrid specific expressed five genes. Here, F: flower buds, DPA: 1 day post anthesis ovule, A: maternal parent, B, and D represent paternal parents of high (H) and low (L) hybrids. [file 12870_2020_2442_MOESM12_ESM.png]

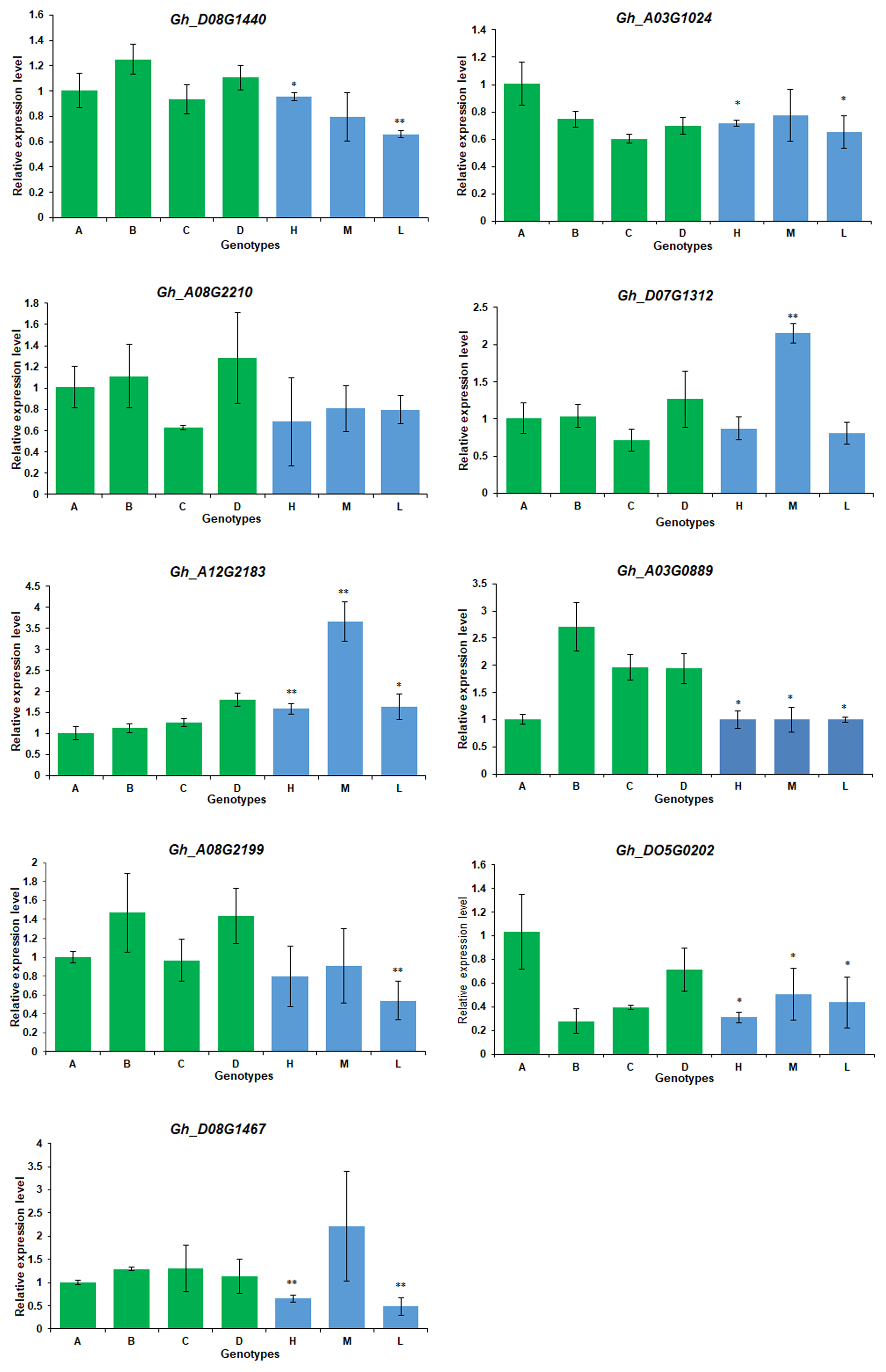

Supplement: Supplementary file 13 — Additional file 13: Figure S13. qRT-PCR of nine selected putative candidate genes. In each figure, H: High, M: Medium, L: Low and A, B, C, and D stands for four inbred parents. The first five genes show expression in flower buds tissue and others represent expression in 1 DPA ovule. * shows a significant difference in hybrids only with their one parent and ** with both parents at p-value 0.05. [file 12870_2020_2442_MOESM13_ESM.png]
