# Supplementary material for: Comparative transcriptome analysis between inbred and hybrids reveals molecular insights into yield heterosis of upland cotton
Source: BMC Plant Biol. 2020 May 27;20:239. doi: 10.1186/s12870-020-02442-z (PMC7251818; doi:10.1186/s12870-020-02442-z)
Supplement: Supplementary file 14 — Additional file 14: Table S1. Mean of mid and better parent heterosis observed for yield traits in three locations and two-year field experimentation. [file 12870_2020_2442_MOESM14_ESM.docx]

| **Hybrids name** | **BN** | | **BW** | | **SCY** | | **LY** | | **LP** | |
| --- | --- | --- | --- | --- | --- | --- | --- | --- | --- | --- |
|  | **MPH** | **BPH** | **MPH** | **BPH** | **MPH** | **BPH** | **MPH** | **BPH** | **MPH** | **BPH** |
| K8-1×851-2 | 2.31 | 4.41 | 9.44 | -0.65 | 9.32 | 2.88 | 9.40 | 0.09 | 0.35 | -3.40 |
| K8-1×A2-10 | 7.07 | 5.67 | 9.58 | 4.21 | 16.15 | 10.12 | 18.26 | 14.25 | 1.86 | -0.03 |
| K8-1×DT-8 | 5.82 | -1.08 | 3.67 | 0.30 | 6.14 | -6.29 | 8.10 | -4.32 | 1.94 | 0.28 |
| K8-1×GC-8 | 11.95 | 10.06 | 6.97 | 2.68 | 18.17 | 11.34 | 21.04 | 15.54 | 2.23 | 2.85 |
| K8-1×RP24-10 | 9.10 | 6.78 | 12.08 | 1.80 | 7.51 | -0.60 | 8.94 | 2.22 | 2.22 | 0.63 |
| K8-1×Z98-15 | 6.28 | -2.27 | 0.04 | 2.35 | 14.38 | 8.12 | 17.66 | 12.47 | 2.77 | 1.27 |
| L28-2 ×RP24-10 | 8.36 | -3.06 | 1.08 | 2.52 | 9.91 | 2.02 | 11.38 | 2.51 | 1.39 | -1.56 |
| L28-2 ×Z98-15 | 3.18 | -3.38 | -0.31 | 0.36 | 8.46 | 2.80 | 13.95 | 8.22 | 4.92 | 1.74 |
| L28-2×851-2 | 5.75 | 7.19 | 12.18 | 1.89 | 12.77 | 4.00 | 15.27 | 0.58 | 2.77 | -4.53 |
| L28-2×A2-10 | 6.21 | 2.50 | 9.92 | 4.54 | 14.91 | 9.15 | 17.75 | 11.47 | 2.53 | -0.54 |
| L28-2×DT-8 | 6.66 | 5.57 | 14.58 | 1.66 | 16.70 | 1.47 | 19.55 | 0.77 | 3.04 | -0.47 |
| L28-2×GC-8 | 5.59 | -3.96 | 3.31 | 2.90 | 16.92 | 9.66 | 19.88 | 9.80 | 2.59 | -1.47 |
| SJ48-1×851-2 | 3.57 | 3.35 | 12.00 | 0.15 | 13.29 | 2.49 | 14.17 | 2.09 | 1.30 | -5.50 |
| SJ48-1×A2-10 | 5.92 | 7.99 | 12.19 | 3.60 | 17.15 | 13.06 | 20.07 | 15.02 | 2.61 | -0.16 |
| SJ48-1×DT-8 | 10.20 | -3.51 | 0.99 | 2.91 | 8.80 | 0.28 | 21.09 | 4.19 | 3.90 | 0.72 |
| SJ48-1×GC-8 | 5.39 | 4.42 | 9.52 | 3.20 | 19.43 | 15.08 | 24.65 | 17.68 | 4.41 | 0.74 |
| SJ48-1×RP24-10 | 9.94 | -6.66 | -1.21 | 3.47 | 14.04 | 8.67 | 16.05 | 9.13 | 2.32 | -0.18 |
| SJ48-1×Z98-15 | 6.46 | 3.25 | 9.51 | 3.00 | 19.89 | 14.07 | 24.25 | 16.24 | 3.72 | 1.01 |
| ZB-1×851-2 | 7.84 | 4.41 | 11.07 | -0.57 | 28.05 | 12.77 | 31.22 | 16.61 | 2.44 | -4.79 |
| ZB-1×A2-10 | 5.93 | -2.91 | 6.71 | -0.49 | 18.66 | 11.88 | 16.11 | 9.11 | -2.56 | -6.02 |
| ZB-1×DT-8 | 8.52 | -5.51 | -1.66 | 4.29 | 30.62 | 18.27 | 34.18 | 20.05 | 2.90 | -0.80 |
| ZB-1×GC-8 | 5.54 | -8.07 | 0.75 | -1.07 | 19.40 | 12.38 | 20.15 | 15.18 | 0.25 | -3.75 |
| ZB-1×RP24-10 | 11.15 | 2.01 | 3.96 | 5.90 | 19.17 | 11.14 | 18.52 | 13.05 | -0.89 | -3.79 |
| ZB-1×Z98-15 | 9.63 | -7.58 | -2.25 | 6.75 | 19.67 | 12.60 | 20.81 | 17.24 | 0.53 | -2.50 |
| Zhong901 -19×851-2 | 10.22 | 2.41 | 8.95 | 5.87 | 16.86 | 4.62 | 18.90 | 6.58 | 2.03 | 0.25 |
| Zhong901 -19×A2-10 | 7.20 | 2.40 | 11.01 | 3.58 | 13.32 | 9.79 | 21.89 | 16.24 | 7.94 | 3.09 |
| Zhong901 -19×GC-8 | 10.73 | -1.11 | 6.47 | 5.69 | 18.19 | 14.12 | 24.17 | 18.88 | 5.02 | 2.42 |
| Zhong901 -19×RP24-10 | 7.81 | 2.42 | 3.88 | 2.83 | 10.00 | 6.12 | 19.28 | 14.12 | 8.53 | 4.69 |
| Zhong901 -19×Z98-15 | 7.50 | -1.31 | 2.56 | 3.77 | 12.46 | 8.69 | 21.63 | 16.54 | 8.19 | 4.48 |
| Zhong901-19×DT-8 | 5.69 | -0.90 | 7.80 | 1.95 | 18.96 | 5.82 | 27.08 | 13.17 | 6.52 | 2.15 |

**Table S1. Mean of mid and better parent heterosis observed for yield traits in three location and two-year field experimentation.**

BN; number of bolls per plant, BW; boll weight, SCY; seed cotton yield, LY; lint yield, LP; lint percentage, MPH; mid parent heterosis, BPH; better parent heterosis. Name in red color represents selected hybrids for transcriptome analysis.
