# Supplementary material for: Comparative transcriptome analysis between inbred and hybrids reveals molecular insights into yield heterosis of upland cotton
Source: BMC Plant Biol. 2020 May 27;20:239. doi: 10.1186/s12870-020-02442-z (PMC7251818; doi:10.1186/s12870-020-02442-z)
Supplement: Supplementary file 15 — Additional file 15: Table S2. Summary of RNA sequencing and mapping for all 63 samples. [file 12870_2020_2442_MOESM15_ESM.docx]

| **Sample** | **Total Raw (base)** | **Valid (base)** | **Valid ratio (reads)** | **Q30%** | **Mapped (%)** | **Unique Mapped**  **(%)** | **Multi Mapped (%)** | **Non-splice (%)** | **Splice (%)** |
| --- | --- | --- | --- | --- | --- | --- | --- | --- | --- |
| **ARL1** | **5.94G** | **5.87G** | **98.8** | **94.4** | **95.2** | **62.9** | **32.3** | **52.1** | **31.0** |
| **ARL2** | **5.35G** | **5.30G** | **99.0** | **94.5** | **95.3** | **61.8** | **33.5** | **51.7** | **30.7** |
| **ARL3** | **7.79G** | **7.71G** | **99.0** | **94.6** | **95.6** | **62.0** | **33.6** | **51.2** | **31.2** |
| **BRL1** | **5.48G** | **5.43G** | **99.1** | **94.6** | **95.5** | **61.5** | **34.0** | **51.0** | **31.2** |
| **BRL2** | **5.33G** | **5.27G** | **98.9** | **95.0** | **95.4** | **61.3** | **34.1** | **51.2** | **30.8** |
| **BRL3** | **7.88G** | **7.77G** | **98.6** | **96.2** | **95.6** | **61.0** | **34.6** | **51.0** | **31.2** |
| **CRL1** | **6.00G** | **5.94G** | **99.0** | **93.7** | **95.0** | **59.1** | **36.0** | **51.2** | **31.0** |
| **CRL2** | **5.36G** | **5.31G** | **99.1** | **93.8** | **95.0** | **59.0** | **36.0** | **51.4** | **30.7** |
| **CRL3** | **6.80G** | **6.72G** | **98.9** | **93.5** | **94.9** | **59.1** | **35.9** | **50.7** | **31.2** |
| **DRL1** | **6.00G** | **5.93G** | **98.9** | **94.4** | **95.2** | **59.8** | **35.4** | **51.2** | **31.3** |
| **DRL2** | **6.84G** | **6.74G** | **98.5** | **95.5** | **95.4** | **60.9** | **34.6** | **50.9** | **31.8** |
| **DRL3** | **5.52G** | **5.45G** | **98.8** | **94.4** | **95.2** | **59.5** | **35.7** | **50.5** | **31.6** |
| **HRL1** | **5.77G** | **5.70G** | **98.9** | **93.6** | **94.7** | **58.3** | **36.4** | **50.2** | **31.4** |
| **HRL2** | **5.95G** | **5.87G** | **98.8** | **94.7** | **95.4** | **59.9** | **35.6** | **50.4** | **31.9** |
| **HRL3** | **6.88G** | **6.79G** | **98.8** | **93.4** | **94.9** | **58.8** | **36.1** | **50.5** | **31.0** |
| **MRL1** | **5.72G** | **5.58G** | **97.5** | **93.7** | **94.3** | **59.5** | **34.8** | **50.6** | **31.0** |
| **MRL2** | **7.92G** | **7.83G** | **98.8** | **93.9** | **95.4** | **60.3** | **35.1** | **51.2** | **31.0** |
| **MRL3** | **6.62G** | **6.52G** | **98.5** | **94.9** | **95.5** | **60.3** | **35.2** | **51.4** | **30.9** |
| **LRL1** | **7.93G** | **7.84G** | **98.9** | **93.8** | **95.1** | **60.0** | **35.1** | **51.1** | **30.9** |
| **LRL2** | **7.31G** | **7.24G** | **98.9** | **92.8** | **94.7** | **58.5** | **36.2** | **51.6** | **30.6** |
| **LRL3** | **5.79G** | **5.72G** | **98.8** | **93.3** | **95.1** | **59.4** | **35.7** | **51.6** | **30.9** |
| **AF1** | **6.30G** | **6.20G** | **98.5** | **94.8** | **95.3** | **60.5** | **34.8** | **52.2** | **30.8** |
| **AF2** | **7.75G** | **7.64G** | **98.6** | **94.0** | **94.5** | **62.0** | **32.6** | **51.5** | **30.9** |
| **AF3** | **6.02G** | **5.95G** | **98.9** | **94.0** | **95.0** | **61.8** | **33.3** | **52.4** | **30.6** |
| **BF1** | **7.41G** | **7.28G** | **98.2** | **94.2** | **95.3** | **63.2** | **32.1** | **52.0** | **31.0** |
| **BF2** | **6.08G** | **5.99G** | **98.5** | **94.2** | **95.3** | **63.2** | **32.1** | **52.3** | **31.0** |
| **BF3** | **7.91G** | **7.79G** | **98.5** | **94.5** | **94.0** | **62.3** | **31.7** | **51.2** | **30.8** |
| **CF1** | **7.02G** | **6.90G** | **98.3** | **94.4** | **88.9** | **58.6** | **30.3** | **48.8** | **28.4** |
| **CF2** | **5.91G** | **5.83G** | **98.6** | **94.1** | **95.1** | **62.5** | **32.7** | **51.6** | **31.5** |
| **CF3** | **5.96G** | **5.88G** | **98.6** | **94.0** | **95.1** | **62.6** | **32.5** | **51.9** | **31.2** |
| **DF1** | **7.60G** | **7.53G** | **99.1** | **94.0** | **95.3** | **62.3** | **32.9** | **51.9** | **31.0** |
| **DF2** | **7.07G** | **7.00G** | **99.1** | **93.8** | **94.7** | **61.6** | **33.1** | **52.5** | **30.5** |
| **DF3** | **7.56G** | **7.49G** | **99.0** | **94.4** | **95.6** | **63.7** | **31.8** | **50.9** | **32.8** |
| **HF1** | **5.99G** | **5.93G** | **99.0** | **93.4** | **92.3** | **60.5** | **31.7** | **51.0** | **29.8** |
| **HF2** | **7.17G** | **7.10G** | **99.0** | **94.1** | **94.7** | **62.4** | **32.3** | **51.7** | **31.5** |
| **HF3** | **5.59G** | **5.53G** | **99.0** | **94.4** | **95.5** | **63.0** | **32.4** | **51.9** | **32.1** |
| **MF1** | **7.44G** | **7.35G** | **98.8** | **94.3** | **95.6** | **63.9** | **31.7** | **52.4** | **31.7** |
| **MF2** | **5.98G** | **5.92G** | **98.9** | **94.2** | **92.3** | **59.8** | **32.4** | **50.4** | **30.8** |
| **MF3** | **5.80G** | **5.75G** | **99.1** | **94.1** | **95.2** | **62.1** | **33.1** | **52.4** | **31.5** |
| **LF1** | **5.76G** | **5.70G** | **99.1** | **94.3** | **95.3** | **62.3** | **33.0** | **51.5** | **32.0** |
| **LF2** | **6.14G** | **6.09G** | **99.2** | **93.8** | **93.4** | **59.9** | **33.5** | **51.3** | **30.5** |
| **LF3** | **6.45G** | **6.40G** | **99.1** | **93.9** | **94.5** | **61.2** | **33.2** | **51.8** | **31.3** |
| **DPAA1** | **7.80G** | **7.72G** | **99.0** | **94.0** | **94.1** | **61.2** | **32.9** | **50.6** | **31.6** |
| **DPAA2** | **7.76G** | **7.68G** | **99.0** | **94.0** | **95.2** | **62.1** | **33.1** | **50.9** | **32.3** |
| **DPAA3** | **6.01G** | **5.96G** | **99.1** | **94.3** | **94.5** | **61.3** | **33.3** | **50.9** | **32.0** |
| **DPAB1** | **7.71G** | **7.63G** | **98.9** | **93.8** | **93.4** | **60.8** | **32.6** | **50.5** | **31.4** |
| **DPAB2** | **5.76G** | **5.70G** | **99.1** | **93.7** | **92.8** | **60.1** | **32.8** | **50.7** | **31.1** |
| **DPAB3** | **5.52G** | **5.40G** | **97.9** | **97.9** | **95.1** | **66.5** | **28.6** | **52.0** | **31.9** |
| **DPAC1** | **5.56G** | **5.47G** | **98.2** | **98.7** | **95.9** | **66.9** | **29.0** | **52.6** | **31.8** |
| **DPAC2** | **6.70G** | **6.58G** | **98.2** | **98.7** | **96.2** | **66.7** | **29.5** | **52.2** | **32.3** |
| **DPAC3** | **5.88G** | **5.78G** | **98.3** | **98.7** | **96.7** | **67.4** | **29.3** | **52.4** | **32.6** |
| **DPAD1** | **6.67G** | **6.52G** | **97.7** | **98.7** | **92.8** | **65.0** | **27.8** | **50.0** | **31.4** |
| **DPAD2** | **5.53G** | **5.22G** | **94.5** | **97.8** | **93.1** | **65.0** | **28.1** | **50.0** | **31.6** |
| **DPAD3** | **6.69G** | **6.56G** | **98.1** | **98.7** | **95.5** | **66.7** | **28.8** | **50.9** | **32.7** |
| **DPAH1** | **7.67G** | **7.55G** | **98.4** | **98.6** | **96.5** | **67.2** | **29.3** | **52.4** | **33.0** |
| **DPAH2** | **7.81G** | **7.67G** | **98.2** | **98.7** | **96.5** | **67.2** | **29.4** | **51.4** | **33.5** |
| **DPAH3** | **7.66G** | **7.54G** | **98.5** | **98.5** | **93.7** | **64.5** | **29.2** | **50.2** | **32.2** |
| **DPAM1** | **5.77G** | **5.67G** | **98.3** | **98.5** | **96.2** | **66.5** | **29.7** | **52.4** | **32.2** |
| **DPAM2** | **6.76G** | **6.64G** | **98.2** | **98.8** | **96.6** | **67.8** | **28.8** | **52.6** | **32.5** |
| **DPAM3** | **6.22G** | **6.11G** | **98.1** | **98.3** | **95.6** | **65.7** | **29.8** | **52.1** | **31.8** |
| **DPAL1** | **7.62G** | **7.48G** | **98.2** | **98.8** | **96.5** | **67.1** | **29.4** | **52.3** | **32.6** |
| **DPAL2** | **7.88G** | **7.68G** | **97.4** | **98.6** | **96.5** | **66.7** | **29.7** | **52.4** | **31.1** |
| **DPAL3** | **5.61G** | **5.47G** | **97.6** | **98.5** | **95.1** | **65.1** | **30.0** | **51.3** | **30.4** |

**Table S2. Summary of RNA sequencing and mapping for all 63 samples**

Here, in Table S2, A, B, C, and D represent four inbred parents and H, M, and L correspond to three hybrids. RL, F, and DPA correspond to leaf, flower bud, and 1 day post anthesis ovule, respectively. The numerical values 1, 2, 3 indicate three different biological replicates.
