# Supplementary material for: Comparative transcriptome analysis between inbred and hybrids reveals molecular insights into yield heterosis of upland cotton
Source: BMC Plant Biol. 2020 May 27;20:239. doi: 10.1186/s12870-020-02442-z (PMC7251818; doi:10.1186/s12870-020-02442-z)
Supplement: Supplementary file 18 — Additional file 18: Table S5. Detailed information of already reported seed cotton yield QTLs used to map DEGs. [file 12870_2020_2442_MOESM18_ESM.docx]

**Table S5. Detailed information of already reported seed cotton yield QTLs used to map DEGs**

| **File** | **Map** | **QTL** | **Chromosome** | **Trait** | **LOD** | **R-sq** | **SimMap** | **Position** | **Left** | **Right** |
| --- | --- | --- | --- | --- | --- | --- | --- | --- | --- | --- |
| Upland | Consensusmap | QTLSCY1.d | 3 | SCY | 2.92 | 16.2 | N | 41.5 | 36.5 | 54 |
| Upland | Consensusmap | QTLSCY1.d | 3 | SCY | 2.92 | 16.2 | N | 41.5 | 36.5 | 54 |
| Upland | Consensusmap | qSY-chr5-1 | 5 | SCY | 3.33 | 6.68 | N | 6.26 | 6.26 | 6.26 |
| Upland | Consensusmap | SCY6a | 5 | SCY | 3 | 1 | N | 12.7 | 0 | 20.77 |
| Upland | Consensusmap | SCY6a | 5 | SCY | 3 | 1 | N | 12.7 | 0 | 20.77 |
| Upland | Consensusmap | qSY-chr5-2 | 5 | SCY | 7.25 | 13.57 | N | 39.08 | 38.9 | 39.6 |
| Upland | Consensusmap | QTLSCY2.d | 6 | SCY | 2.43 | 5.6 | N | 0.29 | 0 | 42.4 |
| Upland | Consensusmap | QTLSCY2.d | 6 | SCY | 2.43 | 5.6 | N | 0.29 | 0 | 42.4 |
| Upland | Consensusmap | qSCY-A6-1 | 6 | SCY | 3.99 | 10.7 | N | 44 | 42.4 | 44.3 |
| Upland | Consensusmap | qSY-A7-1 | 7 | SCY | 5.43 | 10.88 | N | 8.51 | 7 | 17.9 |
| Upland | Consensusmap | qSY-A7-1a | 7 | SCY | 6.54 | 17.9 | N | 14.51 | 7 | 17.9 |
| Upland | Consensusmap | SCY2a | 8 | SCY | 3 | 1 | N | 7 | 6.94 | 12.83 |
| Upland | Consensusmap | SCY2a | 8 | SCY | 3 | 1 | N | 7 | 6.94 | 12.83 |
| Upland | Consensusmap | qSY-chr8-1 | 8 | SCY | 3.66 | 7.5 | N | 38.52 | 34.5 | 44 |
| Upland | Consensusmap | qSY-A9-1 | 9 | SCY | 7.83 | 16.78 | N | 8.91 | 4.9 | 14.8 |
| Upland | Consensusmap | qSY-A9-1a | 9 | SCY | 4.84 | 11.96 | N | 9.91 | 4.9 | 14.8 |
| Upland | Consensusmap | QTLSCY3.d | 9 | SCY | 2.2 | 12.7 | N | 20.01 | 10.01 | 30.01 |
| Upland | Consensusmap | QTLSCY3.d | 9 | SCY | 2.2 | 12.7 | N | 20.01 | 10.01 | 30.01 |
| Upland | Consensusmap | qSY-chr10-1 | 10 | SCY | 3.14 | 5.5 | N | 64.97 | 42.9 | 65.1 |
| Upland | Consensusmap | qSY-chr11-1a | 11 | SCY | 2.78 | 6.08 | N | 0.62 | 0.6 | 1.7 |
| Upland | Consensusmap | qSY-chr11-1b | 11 | SCY | 2.02 | 4.5 | N | 1.73 | 1.7 | 2 |
| Upland | Consensusmap | SCY1a | 11 | SCY | 3 | 1 | N | 3 | 0 | 20.02 |
| Upland | Consensusmap | SCY1a | 11 | SCY | 3 | 1 | N | 3 | 0 | 20.02 |
| Upland | Consensusmap | qSY-chr11-2 | 11 | SCY | 10.15 | 23.13 | N | 73.77 | 63.8 | 81.1 |
| Upland | Consensusmap | qSY-A12-1 | 12 | SCY | 2.59 | 7.52 | N | 0.01 | 0 | 8.5 |
| Upland | Consensusmap | qSY-chr12-2 | 12 | SCY | 4.65 | 7.97 | N | 2.96 | 2.9 | 6.5 |
| Upland | Consensusmap | qSY-chr12-1a | 12 | SCY | 3.59 | 7.62 | N | 14.73 | 14.7 | 25.6 |
| Upland | Consensusmap | qSY-chr12-1b | 12 | SCY | 2.75 | 5.26 | N | 18.73 | 14.7 | 25.6 |
| Upland | Consensusmap | YLD_12 | 12 | SCY | 4.18 | 8.6 | N | 50.5 | 20 | 52 |
| Upland | Consensusmap | SCY4a | 13 | SCY | 3 | 1 | N | 4 | 0 | 5.42 |
| Upland | Consensusmap | SCY4a | 13 | SCY | 3 | 1 | N | 4 | 0 | 5.42 |
| Upland | Consensusmap | SCY2a | 13 | SCY | 3 | 1 | N | 38.8 | 31.8 | 39.96 |
| Upland | Consensusmap | SCY2a | 13 | SCY | 3 | 1 | N | 38.8 | 31.8 | 39.96 |
| Upland | Consensusmap | qSY-chr13-1 | 13 | SCY | 5.44 | 11.54 | N | 125.04 | 120.8 | 125.8 |
| Upland | Consensusmap | qSY-D2-2 | 14 | SCY | 2.69 | 7.15 | N | 8.99 | 0 | 21.89 |
| Upland | Consensusmap | qSY-chr14-1a | 14 | SCY | 5.18 | 12.71 | N | 78.05 | 74.1 | 83.1 |
| Upland | Consensusmap | qSY-chr14-1b | 14 | SCY | 2.22 | 3.64 | N | 83.06 | 83.1 | 83.9 |
| Upland | Consensusmap | YLD_16 | 16 | SCY | 7.25 | 14.4 | N | 40.9 | 41 | 42 |
| Upland | Consensusmap | SCY1.w | 16 | SCY | 3.99 | 8.58 | N | 62.3 | 57.52 | 64.16 |
| Upland | Consensusmap | SCY1.w | 16 | SCY | 3.99 | 8.58 | N | 62.3 | 57.52 | 64.16 |
| Upland | Consensusmap | qSY-D5-1 | 19 | SCY | 2.66 | 7.99 | N | 7.01 | 0 | 15.81 |
| Upland | Consensusmap | SCY7a | 20 | SCY | 3 | 1 | N | 0 | 0 | 14.21 |
| Upland | Consensusmap | SCY7a | 20 | SCY | 3 | 1 | N | 0 | 0 | 14.21 |
| Upland | Consensusmap | YLD_20 | 20 | SCY | 3.78 | 9.3 | N | 11.3 | 7 | 32 |
| Upland | Consensusmap | SCY3a | 22 | SCY | 3 | 1 | N | 4 | 0 | 12.95 |
| Upland | Consensusmap | SCY3a | 22 | SCY | 3 | 1 | N | 4 | 0 | 12.95 |
| Upland | Consensusmap | SCY1a | 22 | SCY | 3 | 1 | N | 18 | 12.95 | 21.06 |
| Upland | Consensusmap | SCY1a | 22 | SCY | 3 | 1 | N | 18 | 12.95 | 21.06 |
| Upland | Consensusmap | qSY-Chr22-1 | 22 | SCY | 3.82 | 27.4576 | N | 75.95 | 75.2 | 76.7 |
| Upland | Consensusmap | SCY5a | 23 | SCY | 3 | 1 | N | 0 | 0 | 4.95 |
| Upland | Consensusmap | SCY5a | 23 | SCY | 3 | 1 | N | 0 | 0 | 4.95 |
| Upland | Consensusmap | qSY-chr23-1 | 23 | SCY | 3.23 | 6.54 | N | 0.01 | 0 | 1.4 |
| Upland | Consensusmap | SCY2.w | 24 | SCY | 2.72 | 10.71 | N | 49.56 | 47.71 | 56.58 |
| Upland | Consensusmap | SCY2.w | 24 | SCY | 2.72 | 10.71 | N | 49.56 | 47.71 | 56.58 |
| Upland | Consensusmap | YLD_26 | 26 | SCY | 5.1 | 10 | N | 19.5 | 0 | 18 |
| Upland | Consensusmap | qSY-Chr26-2 | 26 | SCY | 2.15 | 43.2964 | N | 43.55 | 43.2 | 43.9 |
| Upland | Consensusmap | qSY-chr26-1 | 26 | SCY | 2.35 | 5.24 | N | 63.34 | 61.6 | 64.6 |
