# Supplementary material for: Comparative transcriptome analysis between inbred and hybrids reveals molecular insights into yield heterosis of upland cotton
Source: BMC Plant Biol. 2020 May 27;20:239. doi: 10.1186/s12870-020-02442-z (PMC7251818; doi:10.1186/s12870-020-02442-z)
Supplement: Supplementary file 19 — Additional file 19: Table S6. Number of co-expressed modules and genes for hybrids. [file 12870_2020_2442_MOESM19_ESM.docx]

| **Module name** | **High hybrid** | **Low hybrid** |
| --- | --- | --- |
| Black | 401 | 249 |
| Blue | 1104 | 691 |
| Brown | 1089 | 628 |
| Cyan | 122 | 101 |
| Green | 456 | 309 |
| Green yellow | 182 | 146 |
| Grey | 687 | 244 |
| Grey60 | 55 | 95 |
| Light cyan | 69 | 96 |
| Light green | 46 | 85 |
| Light yellow | 44 | 82 |
| Magenta | 246 | 177 |
| Midnight blue | 115 | 98 |
| Pink | 392 | 185 |
| Purple | 234 | 174 |
| Red | 423 | 252 |
| Royal blue | 34 | 80 |
| Salmon | 124 | 123 |
| Tan | 141 | 138 |
| Turquoise | 1972 | 2880 |
| Yellow | 926 | 322 |
| Darker green | - | 74 |
| Darker gray | - | 68 |
| Darko live green | - | 42 |
| Darko range | - | 63 |
| Dark red | - | 79 |
| Dark turquoise | - | 70 |
| Orange | - | 66 |
| Pale turquoise | - | 45 |
| Saddle brown | - | 52 |
| Steel blue | - | 47 |
| Violet | - | 43 |
| White | - | 59 |
| Total genes | 8862 | 7863 |

**Table S6. Number of co-expressed modules and genes for hybrids**
