# Supplementary material for: Comparative transcriptome analysis between inbred and hybrids reveals molecular insights into yield heterosis of upland cotton
Source: BMC Plant Biol. 2020 May 27;20:239. doi: 10.1186/s12870-020-02442-z (PMC7251818; doi:10.1186/s12870-020-02442-z)
Supplement: Supplementary file 21 — Additional file 21: Table S8. List of gene primers used for qRT-PCR. [file 12870_2020_2442_MOESM21_ESM.docx]

**Table S8. List of gene primers used for qRT-PCR**

| **Gene ID** | **Forward primer** | **Reverse primer** | **Start** | **End** |
| --- | --- | --- | --- | --- |
| Gh_D08G1440 | TGGAGCAACGGAATACACAAC | CATGCATTTGATTTCTTCCCT | 945 | 1090 |
| Gh_A03G1024 | GTCGTAGAAGATGATGGCACT | GGGTAGGAAGAGGAGGAAGGA | 208 | 363 |
| Gh_A08G2210 | GAATTTCGCTAAGCAACAGAT | TCATGCCGCAATTGATGTCCT | 1104 | 1241 |
| Gh_D07G1312 | TCACATGGCTGCTTCTTGAGA | ATACAGGATGCTATTTCTGCC | 1219 | 1344 |
| Gh_A12G2183 | GTTTTGGCAGAAGCGATCCCA | GGGACATTGAAATTGGCTATT | 1246 | 1374 |
| Gh_A03G0889 | GAGACTTTAAGCGCAGAGAGA | ATCCATTCAGTCCTTGCATCC | 1763 | 1895 |
| Gh_A08G2199 | ACAACGTTGAAAAAGGGATGC | TTTTCAGTCTCCAAGTCGACC | 146 | 321 |
| Gh_D08G1467 | AACCCAAGGCAAAATTTCTCG | ATTGATGTCATGAAGTGGTGC | 838 | 970 |
| Gh_D05G0202 | TTTTCACAACGAGGCATTACA | CATGCCGTATAGCGTATCCAT | 1605 | 1756 |
